# Dual‐Responsive Yarn‐Based Artificial Muscles for Adaptive Thermal Management

**DOI:** 10.1002/advs.202521717

**Published:** 2026-01-09

**Authors:** Mengjiao Pan, Xiaohui Zhang, Jinhao Xu, Ziqi Li, Junjie Wang, Qirui Zhang, Yi Liu, Hongfei Yang, Kinor Jiang, Albert Chan, Yucan Peng, Dahua Shou

**Affiliations:** ^1^ Future Intelligent Wear Centre School of Fashion and Textiles The Hong Kong Polytechnic University Kowloon Hong Kong P. R. China; ^2^ Research Centre of Textiles for Future Fashion The Hong Kong Polytechnic University Kowloon Hong Kong P. R. China; ^3^ Research Institute for Intelligent Wearable Systems The Hong Kong Polytechnic University Kowloon Hong Kong P. R. China; ^4^ Department of Building and Real Estate The Hong Kong Polytechnic University Kowloon Hong Kong P. R. China; ^5^ Department of Energy and Resources Engineering College of Engineering Peking University Beijing P. R. China; ^6^ PolyU‐Xingguo Technology and Innovation Research Institute The Hong Kong Polytechnic University Kowloon Hong Kong P. R. China

**Keywords:** adaptive textile, artificial muscle, dual responsiveness, thermal management

## Abstract

In the face of global extreme weather and a changing climate, the development of adaptive textiles that provide thermoregulation by altering their structure has become increasingly critical. Such textiles require artificial muscles capable of fast, reliable motion to effectively respond to fluctuating environmental conditions. However, moisture‐responsive yarn‐based muscles, while promising candidates, are hindered by slow and uncontrollable actuation, limiting their practical application. Inspired by the conscious movement of skeletal muscle, we present a hybrid yarn‐based artificial muscle (HYAM) that couples moisture actuation with electrothermal recovery. This design achieves a 128% increase in actuation stroke and a 136% increase in speed compared with pristine yarn‐based muscles. Electrothermal recovery controllably shortens recovery and full‐cycle times by 91% and 83%, respectively. Inspired by spindle‐driven gates, HYAM flips dual‐mode radiative curtains to switch between warming and cooling, showing promise for intelligent personalized thermal management.

## Graphical abstract

Inspired by the conscious movements of skeletal muscle, a novel hybrid yarn‐based artificial muscle (HYAM) coupling moisture actuation with controllable recovery is developed. This unique design increases actuation stroke by 128% and shortens recovery time by 91%. This unique design enhances the actuation stroke by 128% and reduces the recovery time by 91%. It is successfully applied to adaptively flip a dual‐mode radiation fabric, enabling switchable warming and cooling functions for all‐weather smart thermal management textiles.

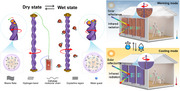

## 1 Introduction

In the face of extreme weather patterns and a rapidly changing climate, smart textiles have garnered considerable attention for their potential to enhance energy efficiency and adaptability. Among these, stimuli‐responsive textiles triggered by temperature, light, electricity, magnetism, or moisture stand out as promising solutions. Among various stimuli‐responsive artificial muscles, such as electroosmotic muscles [1, 2, 3] or shape memory alloys [4], moisture‐responsive muscles offer distinct advantages, particularly in adaptive thermal management for smart wearables and textile engineering [5]. For instance, although the electroosmotic muscles offer rapid responsiveness, the complex integration of electrodes and electrolytes brings challenges for structural design and application in textile systems. However, moisture‐responsive muscles exhibit inherent compatibility with textiles and high sensitivity to moisture, a stimulus that is ubiquitous both in the human body and the surrounding environment. The moisture‐responsive behavior primarily arises from the adsorption and desorption of moisture or solvents by hygroscopic materials, leading to volumetric changes in the material structure [6, 7]. Furthermore, their softness, flexibility, long‐term stability, and lightweight nature of moisture‐responsive muscles make them ideal candidates for textile integration [8]. Recent studies have demonstrated that these muscles can achieve outstanding responsiveness, energy efficiency, and practicality, making them particularly attractive for applications in self‐regulating thermal management textiles and sustainable energy solutions [9, 10, 11, 12].

Several innovative structures of stimuli‐responsive artificial muscles have been designed for smart textiles [13, 14, 15]. Many of these structural principles are also broadly applicable to moisture‐responsive muscles. Among these, yarn‐based artificial muscles are particularly promising, as yarns that are the basic building blocks of textiles can be transformed into artificial muscles by inserting and fixing twists. These muscles can be seamlessly integrated into smart textiles with tailored deformation behaviors. Additionally, their scalability, structural flexibility, and modular design allow for efficient manufacturing and easy customization, ensuring adaptability to diverse applications and expanding their potential in smart textile innovations.

Despite recent progress, moisture‐responsive yarn‐based artificial muscles (MYAM) still face significant challenges. Current designs often suffer from slow and uncontrollable actuation, limiting their practical applications and hindering commercialization. A key issue is the slow recovery actuation observed in most MYAMs [7, 16, 17]. The recovery time is typically much longer than the actuation time, leading to low actuation frequency (the reciprocal of the duration for one absorption–desorption cycle, which also refers to the number of actuation cycles per unit time) [18]. For instance, after only 5 s of wetting, the MYAMs may require up to 115 s to return to their initial state [19]. While high hydrophilicity enhances moisture absorption, it also slows the evaporation of water molecules, significantly delaying recovery. Additionally, the complexity of moisture fluctuations in real environments makes precise and consistent actuation difficult to achieve. Methods such as electrochemical oxidation have been explored to enable controllable electrothermal recovery [20]. However, these approaches are often complex and applicable to only a limited range of materials. Therefore, developing an effective and versatile strategy to improve both actuation frequency and precision is essential. Such a strategy would preserve the inherent strengths of MYAMs while enabling timely feedback and precise control for advanced applications.

**FIGURE 1:**
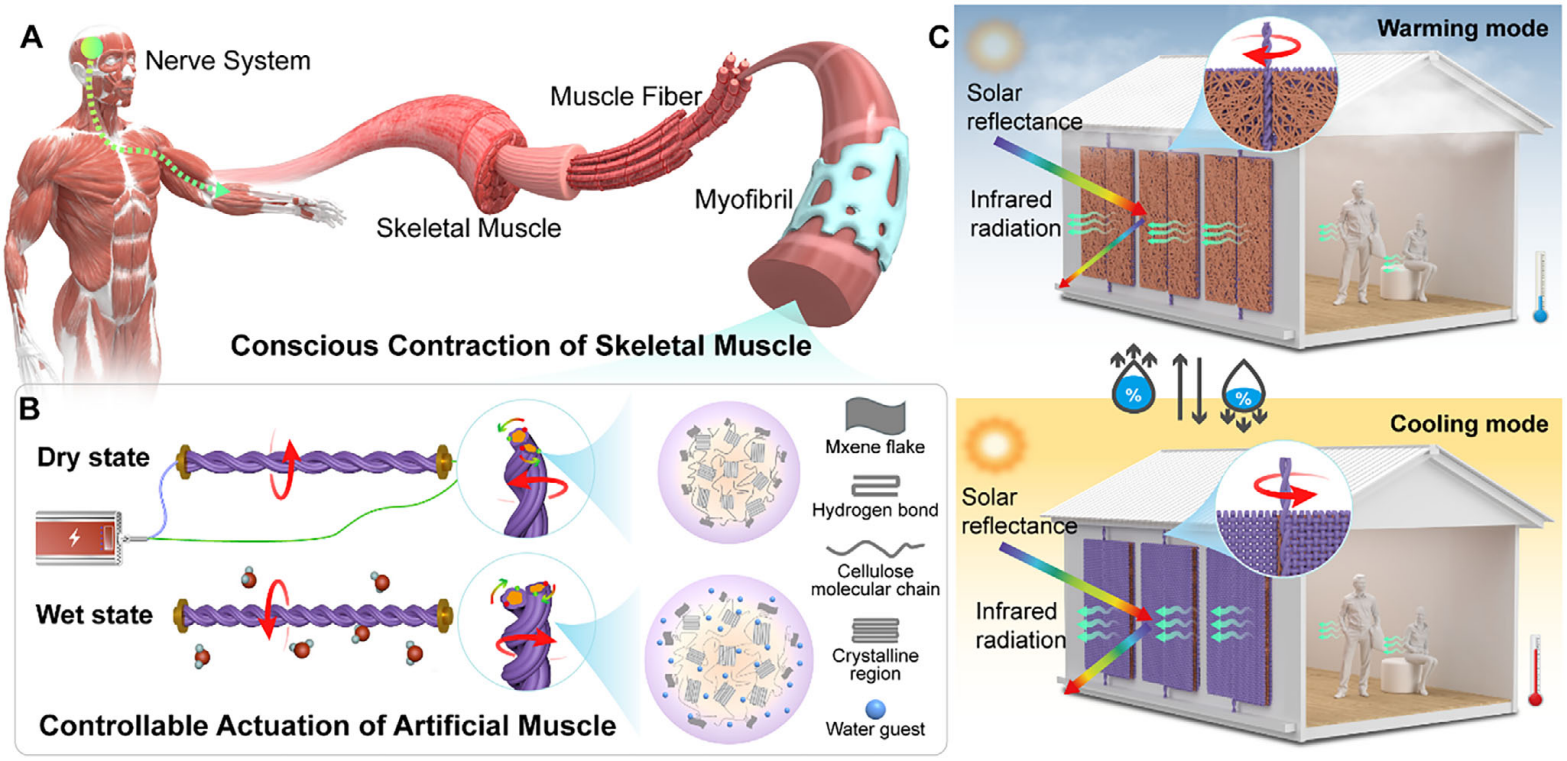
Concept and mechanism of hybrid yarn‐based artificial muscles (HYAMs). A) Bioinspired structural organization and conscious movement of skeletal muscle, consisting of myofibrils. B) Design and actuation mechanism of HYAM with moisture (passive) and electrothermal (active) actuation. C) Application in dual‐mode radiation fabric (DRF): the fabric flips to a warming mode in cool and humid conditions and recovers to a cooling mode in hot and dry conditions, enabling adaptive and sustainable thermal management.

As shown in Figure 1A, skeletal muscle is a voluntary muscle whose movement is consciously controlled via stimulation from the somatic nerve system. Upon receiving contraction signals from the nervous system, a chemical reaction occurs, causing the myofibrils to shorten linearly. Inspired by this mechanism, we develop a novel HYAM capable of both moisture actuation and controllable recovery, addressing the critical issue of slow and uncontrollable recovery in current designs (Figure 1B). The HYAM actuates upon moisture absorption, as water guests bind to hydrophilic components (cellulose and MXene), causing fiber volume expansion. The integrated MXene conductive network enables rapid and controllable recovery via electrical stimulation, mimicking the conscious movement of skeletal muscle. Compared with pristine muscles, the HYAM enhances actuation stroke and speed by 128% and 136%, respectively. Electrothermal recovery also reduces the recovery and full cycle time by 91% and 83%, respectively. This combination of fast, reversible actuation and recovery enables HYAM to flip a DRF for adaptive and switchable thermal management (Figure 1C). For cooling mode, the DRF turns to one side with high solar reflectivity and mid‐infrared emissivity to provide radiative cooling. The DRF can be flipped to another side with high solar absorbance and low mid‐infrared emissivity for warming. This innovative strategy opens new opportunities for all‐weather, sustainable smart thermal management textiles.

## 2 Results and Discussions

### 2.1 Fabrication and Torsional Actuation of Yarn‐Based Artificial Muscles

**FIGURE 2:**
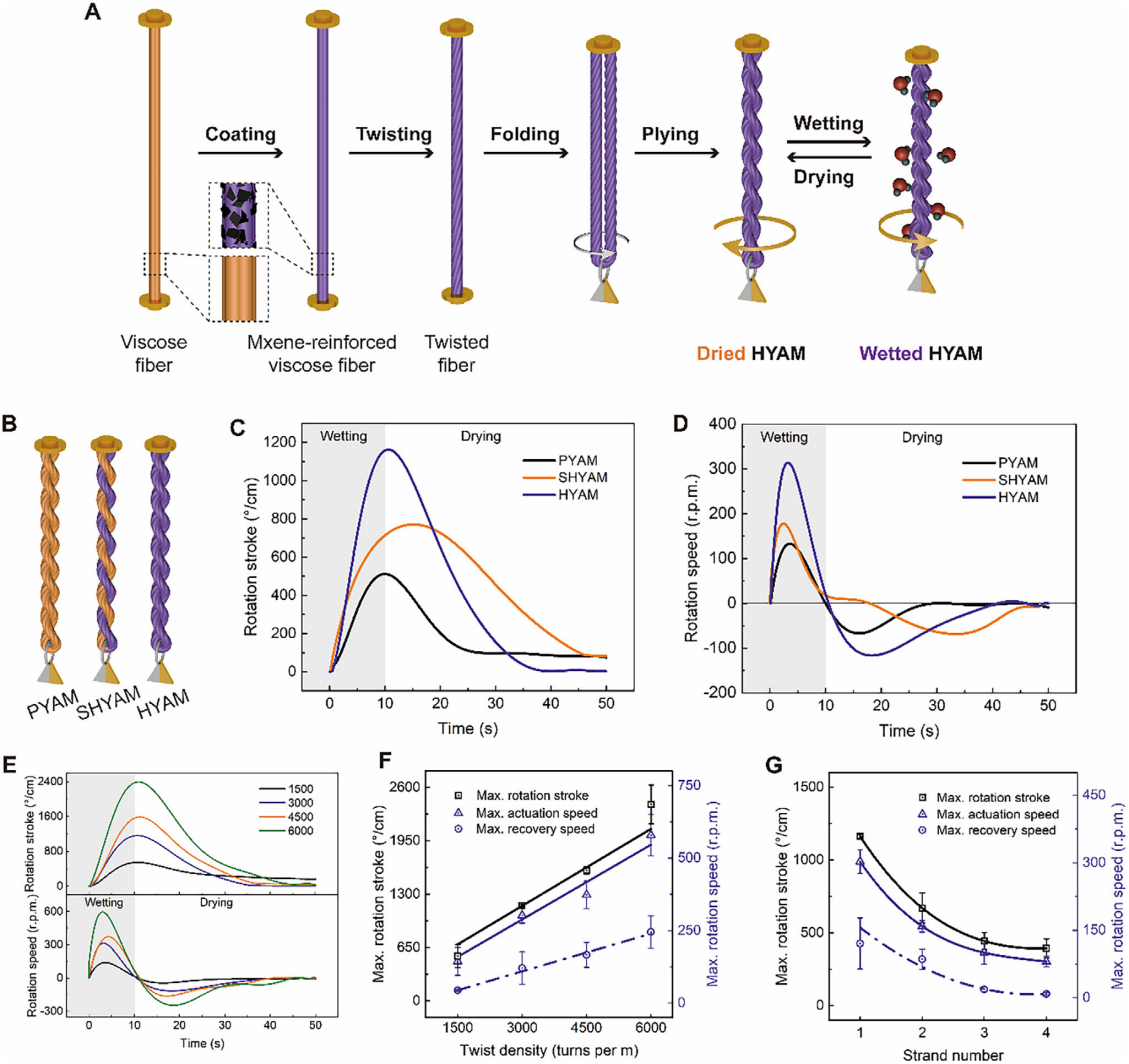
Fabrication process and torsional actuation performance of yarn‐based artificial muscles. A) Schematic illustration of the fabrication of a HYAM. B) Schematic illustration of the structure of pristine (PYAM), semi‐hybrid (SHYAM), and hybrid (HYAM) yarn‐based artificial muscles. A uniform twist density of 3000 tpm is applied to these three yarn‐based muscles. Time dependence of C) rotation stroke and D) rotation speed for one sorption–desorption cycle for the three yarn‐based muscles. E) Time dependence of rotation stroke and speed for one sorption–desorption cycle for HYAM with twist densities of 1500, 3000, 4500, and 6000 tpm. F) Effect of twist density on the maximum rotation stroke, maximum actuation speed, and recovery speed of HYAM. G) Effect of strand number on the maximum rotation stroke, maximum actuation speed, and recovery speed of HYAM, where each HYAM has a twist density of 3000 tpm.

In this work, we design torque‐balanced yarn muscles that remain mechanically stable by attaching a load at the midpoint, and the two segments twist together automatically. As shown in Figure 2A, viscose fibers with a high water retention value [21] are drawn through a concentrated gel‐state Ti_3_C_2_T_x_ MXene dispersion (100 mg/ml, monolayer structure), enabling MXene flakes to adhere to the fiber surface and form a continuous conductive network. Driven by its superior conductivity over the multilayer structure [22], monolayer MXene is selected to achieve better electrothermal recovery. Then, the fibers are twisted in the direction of Z (a right‐handed twist), which further ply in the direction of S (a left‐handed twist), forming a HYAM labeled as Z chirality. This fabrication process is user‐friendly, utilizing cost‐effective and readily available materials. The viscose core provides primary moisture‐responsive torsional energy and is reinforced by a hydrophilic MXene sheath. Meanwhile, the conductive MXene can create controllable and simultaneous torsional recovery through Joule heating.

When absorbing water molecules, each fiber of HYAM untwists due to its volume expansion, leading to an increased level of plying between the two fibers, as the directions of plying and twisting are opposite. HYAM provides enhanced actuation when absorbing moisture, with controllable and simultaneous electrothermal recovery during moisture desorption. This is attributed to the high hydrophilicity and excellent conductivity of MXene networks on fiber surfaces [23]. For comparison, we fabricate SHYAM and PYAM by half‐coating and non‐coating, respectively (Figure 2B). Figure S1 shows representative images of PYAM, SHYAM, and HYAM, along with their pristine and coated surface morphologies.

The investigated HYAM (10 cm in length, 116 µm in diameter, 321 µg in weight) is fabricated from one fiber twisted with 3000 turns per m (tpm) in the direction of Z and plying together. The same parameters are used for PYAM and SHYAM. Unless otherwise noted, the yarn‐based muscles in this work are inserted into 3000 tpm with a Z chirality. In the torsional actuation test, a 12.1 mg paddle is attached at the end of the yarn‐based muscle to magnify the actuation and recovery visually. The yarn‐based muscle is actuated to untwist when deionized moisture is delivered for 10 s at a speed of 0.1 g s^−1^. Then it is dried in an environmental chamber (30–50% RH, 25°C) for 40 s torsional recovery. Figure 2C compares the time dependence of paddle rotation stroke for a PYAM, SHYAM, and HYAM undergoing one complete reversible cycle of moisture actuation and recovery. The HYAM (1163°/cm) enhances the Max. rotation stroke by 51% and 128% compared to SHYAM (771°/cm) and PYAM (511°/cm). The large rotation stroke can be ascribed to the MXene‐reinforced HYAM with numerous hydrophilic groups (─OH and ─O─), which can enhance moisture sorption and volume expansion capacity for the muscle [24].

Furthermore, during 40 s drying, HYAM can almost recover to its initial state while the other two muscles lose a rotation stroke of about 80°/cm. The outer diameters of the single fiber and HYAM increase (from 58 to 77 µm, and from 145 to 157 µm, respectively) due to the moisture wetting, actuating the untwisting of yarn‐based muscles, which can almost return to their initial state (58 and 142 µm, respectively) by drying (Figure S2). The HYAM accordingly shows a larger rotation speed (peak value of 314 and 116 rotations per minute (r.p.m.), respectively) when absorbing and desorbing moisture, compared with PYAM (peak value of 133 and 67 r.p.m., respectively) and SHYAM (peak value of 178 and 68 r.p.m., respectively) (Figure 2D). The faster rotation is expected due to the more abundant hydrophilic groups on the surface of HYAM, which accelerate the combination with water molecules from the microenvironment. Owing to the coated MXene flakes, HYAM provides increased failure strain and breaking stress than PYAM and SHYAM (Figure S3). These results indicate that coating MXene flakes on the viscose yarn‐based muscles effectively enhances their torsional actuation and mechanical strength [25].

The rotation of yarn‐based muscles strongly depends on the inserted twist density. With the increasing twist inserted into the HYAM, its helical angle increases as expected, which agrees well with the calculated values (Figure S4). A larger helical angle indicates that larger torsional energy is potentially stored in HYAM. Therefore, the rotation stroke and speed of HYAM increase while the inserted twist density increases (Figure 2E). The Max. rotation stroke can reach 2394°/cm, meanwhile, the Max. rotation and recovery speed are about 595 and 246 r.p.m., respectively, in the case of HYAM with 6000 turns inserted per meter. Figure 2F further reveals that the rotation stroke and speed are positively linearly correlated with twist density. In each case of different twist densities (Figure S5), HYAM provides a lower Max. recovery speed than that during wetting. This may come from the fact that it is difficult for absorbed moisture to evaporate from fibers with good hygroscopicity, especially in a natural environment. To explore the scalability of HYAM, we fabricate and compare the yarn‐based muscles with two, three, and four‐strand fibers (Figure S6; Figure 2G). A polynomial relationship exists between rotation stroke, speed, and strand number. The HYAM fabricated with fewer fibers always behaves better within short‐term moisture exposure, which can be related to less friction between fibers during actuation and recovery.

### 2.2 Fast Electrothermal Recovery of Dual‐Responsive Yarn‐Based Artificial Muscles

**FIGURE 3:**
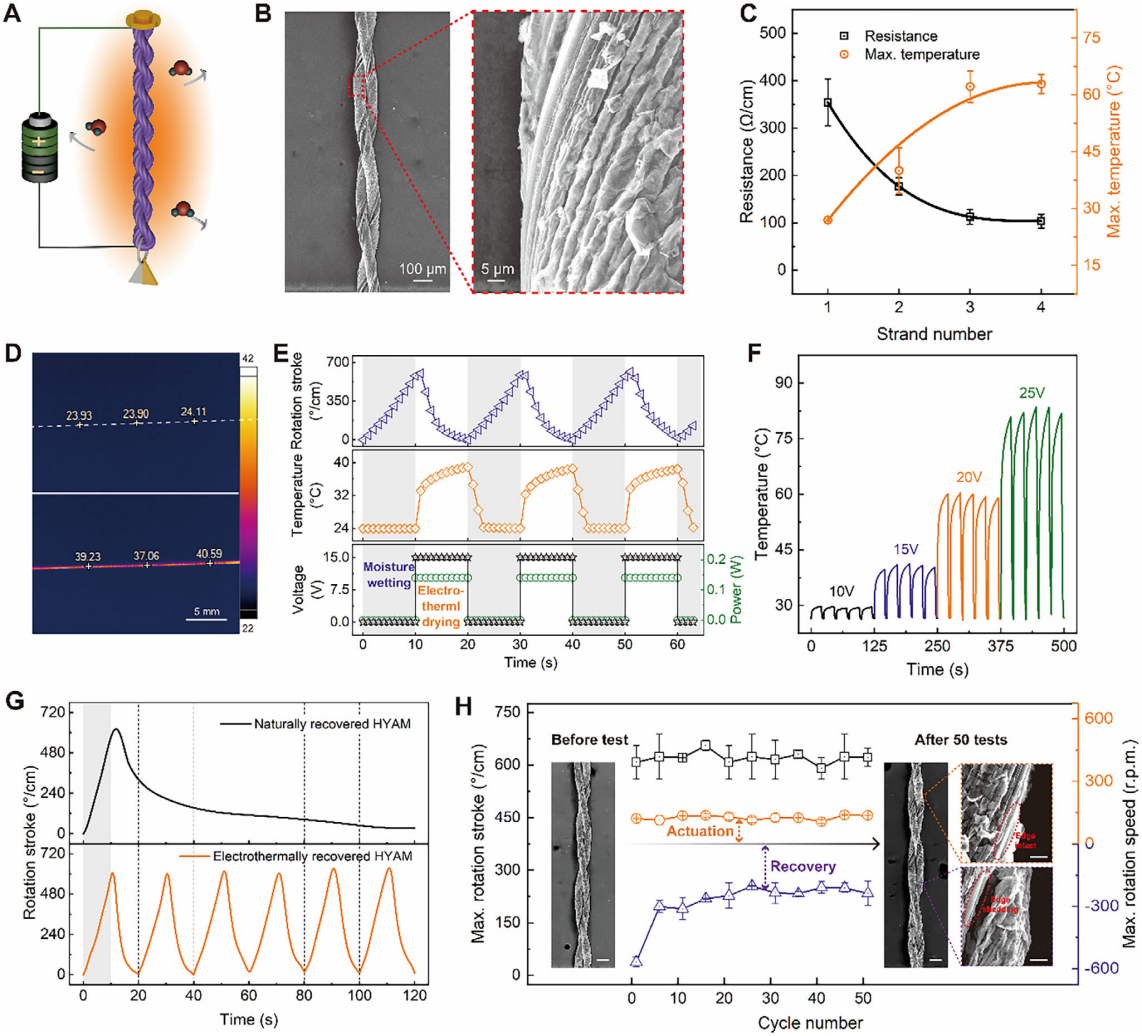
Fast electrothermal recovery of HYAM. A) Schematic illustration of electrothermal recovery for HYAM. B) SEM images of HYAM (left) with large MXene flakes on the surface (∼100 mg/ml, ∼0.5–5 µm in diameter) (right). C) Effect of fiber strand on the resistance and the Max. heated temperature under 15 V of HYAM. D) Thermal images of two‐strand HYAM at a direct voltage of 0 V (top) and 15 V (bottom), respectively. The HYAM is coated with large MXene flakes once. E) Time dependence of rotation stroke and power consumption on the applied voltage, and heated temperature during moisture wetting (gray region) and electrothermal drying (white region) cycles. F) Time dependence of heated temperature at repetitively applied voltages of 10–25 V. The voltage on and off durations within one cycle are about 20 and 5 s, respectively. G) Comparison of rotation stroke recovery between naturally (top) and electrothermally recovered HYAM during the sorption–desorption cycle(s). H) Maximum rotation stroke, actuation, and recovery speed during 50 sorption–desorption cycles. Inset: HYAM before and after 50 cycle tests (Scale bars = 100 and 10 µm for low and high magnification SEM images, respectively).

Joule heating enables the controllable and efficient recovery of HYAM during moisture desorption. As shown in Figure 3A, the top and bottom ends of HYAM are connected to a power supply using silver‐coated nylon wires. When voltage is applied, the MXene conductive network on the fiber surface (Figure 3B) distributes heat across the yarn, causing absorbed water molecules to evaporate quickly. This accelerates the initialization of yarn volume, allowing the HYAM to rapidly retwist and generate reverse torsional actuation. Figure 3C demonstrates that increasing the number of strands reduces resistance and raises the maximum heating temperature due to the larger surface area for conductive flakes. To balance performance and safety, a two‐strand HYAM is adopted for further studies. Additionally, tests on MXene flake size (Figure S7) reveals minimal impact of smaller flakes on performance. The MXene sheath thickness is directly determined by the coating time. Specifically, a single coating results in a thickness of ∼129 nm, which increases to ∼243 nm by coating twice and further to ∼359 nm by three times. Increased coating time improves the electrothermal effect but slightly reduces recovery speed (Figure S8). Therefore, unless otherwise mentioned, HYAM in this study is coated once with large MXene flakes. The effect of ambient temperature and actuation humidity are systematically explored (Figure S9), demonstrating that increasing the humidity difference from 15% RH to 45% RH at various temperatures, HYAM exhibits enhanced rotation stroke and Max. speed. The HYAM performs effectively under a broader environmental condition, laying a foundation for its application in dynamic environments.

Thermal imaging (Figure 3D) shows that applying 15 V raises the temperature from 24°C to about 39°C within 20 s, demonstrating the rapid electrothermal recovery capability of HYAM. Figure 3E illustrates the time‐dependent rotation stroke and power consumption of HYAM during reversible moisture wetting and electrothermal drying cycles. Within 10 s of wetting, the HYAM quickly rotates, achieving a stroke of approximately 601°/cm. When a direct voltage of 15 V is applied, the HYAM heats to 39°C, initiating reversible rotation and returning to its original state. Figure 3F investigates the electrothermal effect under different voltages, showing that the temperature rises and stabilizes during cycles. The energy consumption for 10 s electrothermal drying per cycle is further calculated (Figure S10). A small temperature increase (26–30°C) occurs under 10 V, while higher voltages of 15, 20, and 25 V result in maximum temperatures of approximately 40, 60, and 81°C, respectively.

To demonstrate rapid electrothermal recovery, Figure 3G compares naturally dried and electrothermally dried HYAM (Movie S1). Naturally dried HYAM at room temperature (26°C) is significantly slower, completing only one sorption–desorption cycle in 120 s, whereas electrothermally dried HYAM at ∼40°C completes six cycles in the same time, reducing cycle time by 83%. Although the energy efficiency of electrothermal recovery is limited by Joule heat loss and thermoelectric conversion efficiency, the actuation frequency is significantly improved. Over 50 wetting and electrothermal drying cycles (Figure 3H), HYAM exhibits relatively stable torsional actuation and recovery performance. The maximum rotation stroke shows minimal variation (within 10%), and the actuation speed remains steady at 120–130 r.p.m. SEM images confirm the yarn structure remains largely intact before and after testing. However, the maximum recovery speed drops from 568 to 260 r.p.m. within the first 15 cycles, stabilizing at around 240 r.p.m. This reduction is attributed to partial shedding of MXene coatings, as seen in high‐magnification SEM images (right in Figure 3B). Despite minor coating loss, most remain intact, slightly reducing conductivity and Joule heating, which slows water desorption compared to a new HYAM. To assess the long‐term stability of the HYAM under realistic, fluctuating humidity conditions, its actuation performance is evaluated during 500 humidity‐electrothermal cycles (Figure S11A). The resistance of the HYAM increases slightly due to the partial shedding of MXene at the edges (Figure S11B), leading to a minor decrease in its Max. recovery speed. However, the effective retention of the viscose core and the majority of the MXene coating, along with the intact structure of the HYAM, ensure stable Max. rotation stroke and actuation speed. These results demonstrate the application stability of the HYAM in real‐world environments with dynamic humidities. Furthermore, the rotation stroke and speed remain stable after 10 000 stretching cycles (Figure S12), showcasing HYAM's excellent tensile durability.

### 2.3 Theoretical and Simulation Analysis

For an in‐depth understanding of torsional behaviors, theoretical and simulation analysis of yarn‐based muscle is conducted to explain its torsional mechanism. The HYAM is fabricated from a single torsional fiber, then folded and plied in the middle. Physically, the torsional actuation and recovery are driven by the morphology variation of fibers [26], mainly concerning the helical angle (αf), which can be approximately calculated by Equation (1),

(1) $$\alpha_{f}=tan^{-1}\left(\pi dT\right)$$

where d is the diameter of the HYAM and T is the inserted twist per fiber length. The helical angles observed from the SEM images for HYAM with different inserted twists (Figure S5) agree well with the calculated results (Figure S4) using Equation (1).

**FIGURE 4:**
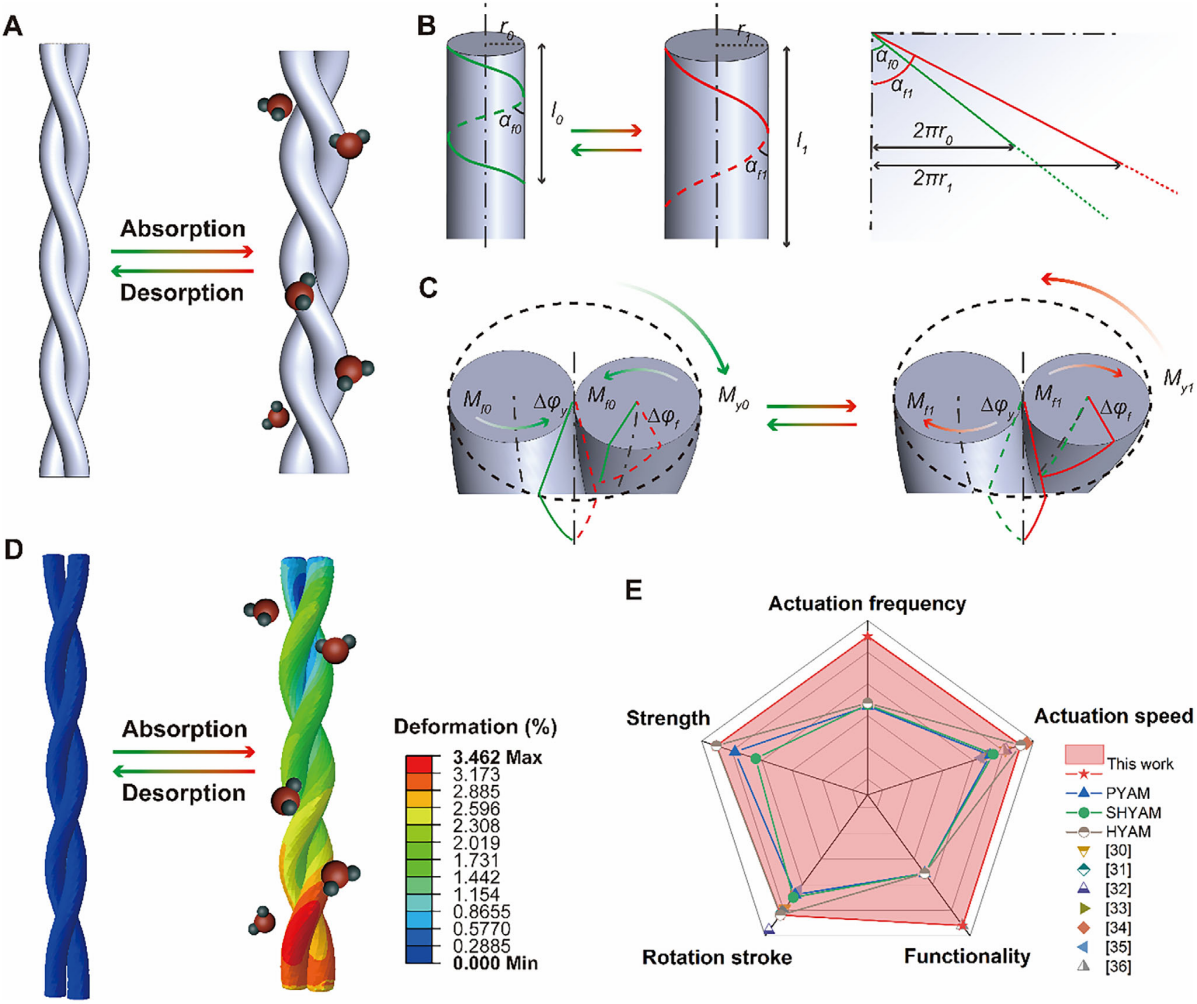
Mechanism and theoretical analysis for yarn‐based artificial muscles. A) Geometric models of coaxial spiral fibers in response to moisture absorption and desorption. B) Spatial spiral motion and planar illustration of each fiber during moisture absorption and desorption. C) Torque behaviors and physical motions of yarn‐based muscles. D) Finite element simulation results of the moisture‐mechanical energy conversion in yarn‐based muscles. E) Comparisons between the HYAM and other yarn‐based muscles.

The plying of yarn‐based muscle increases in the opposite direction to the single fiber untwisting, as a result of the increased helical angle during water molecular absorption. The plying decreases and the torsion recovers to the beginning when desorption occurs (Figure 4A). Upon the water absorption and desorption, the geometric relationships of torsional fibers in one unit (Figure 4B) can be explained by Equations (2) and (3).

(2) $${\left(\frac{s}{t}\right)}^{2}={\left(\frac{l}{t}\right)}^{2}+{\left(\pi d\right)}^{2}$$

(3) $$\sin\alpha_{f}=\frac{\pi d}{s/t}\;,\;\tan\alpha_{f}=\frac{\pi d}{l/t}$$

Thereafter, the spiral path of fibers can be depicted as Equation (4).

(4) $$\delta t=\frac{\delta s}{sin^{2}\alpha_{f}}-\delta \frac{d}{2}-\frac{\delta l}{tan^{2}\alpha_{f}}$$

where δt, δ$\frac{d}{2}$, and δl refer to the relative changing rates of the twist, length, and radius of fibers, respectively. Thereby, the fibers' movement trajectory (δs) during water molecular adsorption and desorption can be obtained. Based on the assumption of relatively small δl and δ$\frac{d}{2}$ upon wetting, the increased radius of fiber drives the decrease of δs, thus inducing the actuation of fibers.

Each fiber's torsion further drives the yarn's torsion during wetting and drying. In ideal conditions, ignoring the energy consumption or mass changes, the torsional energy is converted between fibers and yarn‐based muscle. With the decreasing fiber torsional energy, the HYAM stores increased energy, and the torsional energy in this system can be expressed as Equation (5).

(5) $$\omega M_{f}\Delta \varphi_{f}=-M_{y}\Delta \varphi_{y}$$

where ω is the fiber strands in yarn‐based muscle; M_f_ and M_y_ are the torsional moments of fibers and yarn‐based muscle, respectively; ∆φ_f_ and ∆φ_y_ are the arcs of fibers and yarn‐based muscle, respectively. As illustrated in Figure 4C, the number and direction of twist changes for yarn‐based muscle can be understood according to the variation of each fiber. Theoretically, the rotation stroke of muscle increases as the fiber twist and helical angle increase, showing consistency with our experimental results and previous works [27, 28].

(6) $$\Delta T=-T_{0}\frac{\Delta D}{D_{0}}$$

where ∆T and ∆D are the twist variation of the yarn‐based muscle and the diameter variation of a single fiber under various wetting states, respectively; T_0_ and D_0_ are the twist density of the yarn‐based muscle and the diameter of the fiber under dry state, respectively. As exhibited in Figure S13, the measured rotation strokes of muscle with different twists are compared to the calculated values based on measured geometric parameters. High consistency can be observed, which verifies the good accuracy of the geometric model and theoretical formula. Besides, the measured values are relatively lower than the calculated ones in all cases. This is attributed to the dissipation of frictional energy between fibers in real tests.

Furthermore, we investigate the deformation of yarn‐based muscle using finite element analysis (FEA). A spiral model is established, and one end of the yarn is fixed while another is free. The process of yarn deformation caused by moisture absorption of fibers is predicted, and more details about the simulation are shown in the Supporting Text. The results (Figure 4D) reveal that the deformation of volume changes of fiber, in agreement with previous ideas [29]. This reversible process can also be vividly observed at the micrometer scale (Movie S2). The anisotropic viscose fibers expand horizontally and contract longitudinally upon wetting, triggering the displacement in length and the rotation on the plane for the yarn‐based muscle. Additionally, the muscle can also perform reversibly when desorption occurs. From the simulations, the reversible actuation of yarn‐based muscle derives from the volume changes of fibers caused by moisture condition changes and its plying structure.

Unlike most yarn‐based muscles, the HYAM developed in this work responds to moisture and electric heating, accelerating the desorption process during drying. This dual responsiveness increases the actuation frequency (the reciprocal of the total cycle time for rotation and recovery, that is, the number of actuation cycles per unit of time) and thereby enhances overall muscle functionality. The HYAM achieves increases in actuation stroke and speed of up to 128% and 136%, respectively. Concurrently, electrothermal recovery reduces the recovery and full‐cycle times by up to 91% and 83%, respectively, while the mechanical strength is improved by up to 35%. Consequently, by combining enhanced actuation performance with superior mechanical robustness, the HYAM delivers all‐around good performances (Figure 4E; Tables S1 and S2) [30, 31, 32, 33, 34, 35, 36].

### 2.4 Application in Adaptive Radiation Regulation

**FIGURE 5:**
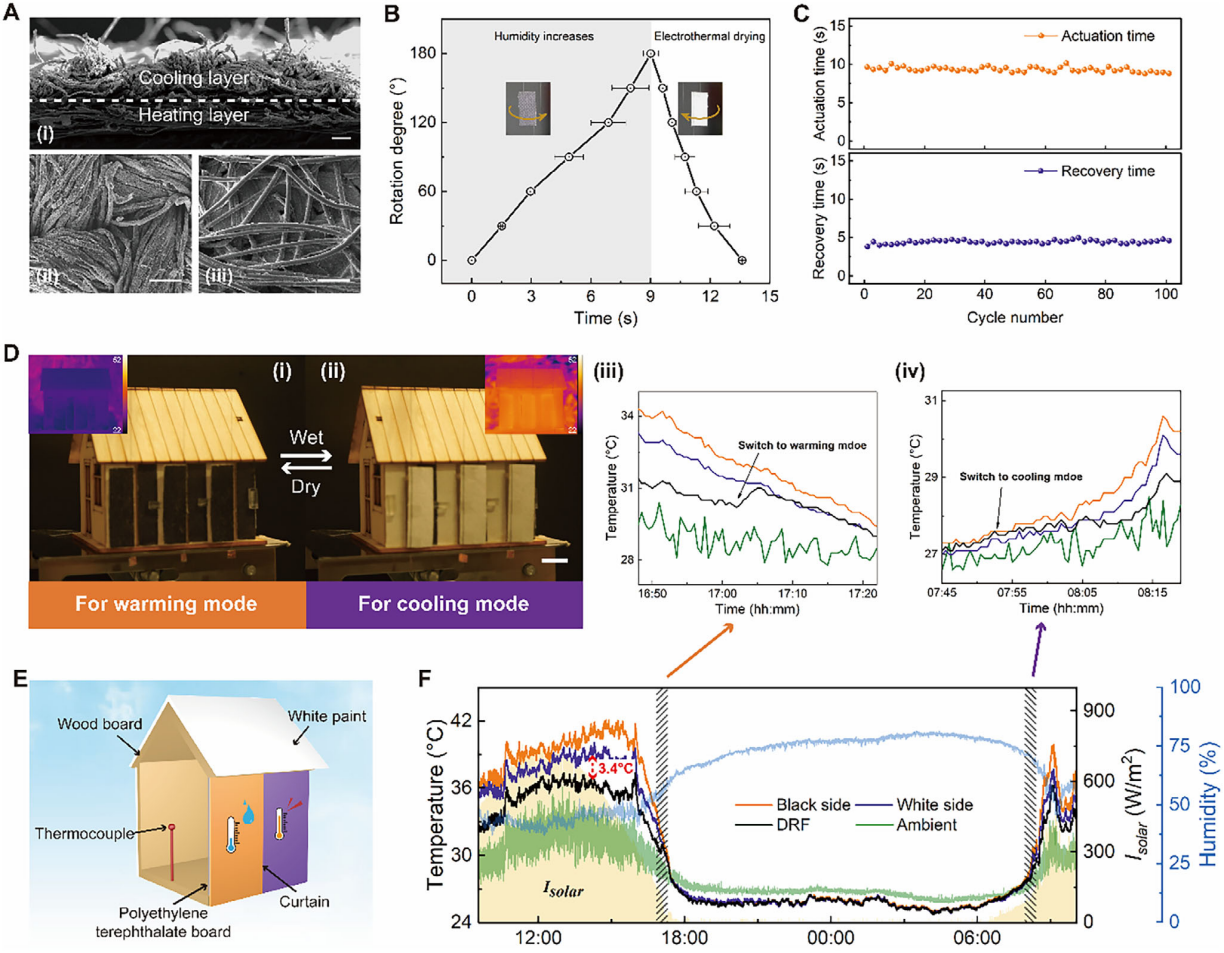
Application of HYAM in adaptive thermal management. A) Structure and scanning electron microscopy images of the cross‐section i), cooling layer ii), and heating layer iii) of DRF (Scale bars = 100 µm). B) Time dependence of fabric rotation degree for one sorption–desorption cycle. C) The actuation and recovery time for the fabric to rotate 180° during 100 sorption–desorption cycles. D) Moisture‐responsive thermal management curtains (scale bars = 1 cm) for warming in humid environments i) and cooling in dry environments (i). Practical application testing, demonstrating the significant warming iii) and cooling iv) effect of adaptive thermal management (October 12–13, 2024, Hong Kong, China). E) Schematic illustration of model house field test setup. F) 24 h field test tracking solar radiance, ambient temperature, humidity, and temperature inside the model house (October 12, 2024, Kowloon, Hong Kong, China).

Inspired by the “gate” opening and closing driven by the “spindle” rotation, the reversible rotation of HYAM can flip a DRF to achieve adaptive and switchable warming and cooling (Figure S19). We fabricate a DRF with a double‐sided design, that is a black‐cotton side coated with MXene flakes for warming, and a white‐cotton side coating of polydimethylsiloxane (PDMS) and TiO_2_ for radiative cooling (See details in Experimental Section and Figure S14). By hot pressing, these two layers are tightly stacked (Figure 5A‐i). As illustrated in the SEM images, the cooling layer (Figure 5A‐ii) homogeneously contains amounts of PDMS and TiO_2_ nanoparticles, evidenced by the distribution of element composition (Si, Ti) from energy dispersive X‐ray spectroscopy (EDX) results (Figure S15). In addition, MXene flakes embedded in the heating layer (Figure 5A‐iii) have a diameter of 0.5–5 µm, and the EDX elemental mapping confirms the even distribution of the MXene coating (Ti, F, Cl) (Figure S15). The DRF functions with high solar absorbance ($\bar{\alpha }$∼72%) and low mid‐infrared (MIR) emissivity ($\bar{\varepsilon }$∼31%) in warming mode, meanwhile with high solar reflectivity ($\bar{\gamma }$∼91%) and MIR emissivity ($\bar{\varepsilon }$∼89%) in cooling mode (Figure S16). Outdoor thermal measurement using customized experimental devices (Figure S17) show that the DRF in warming mode performs ∼10°C above the black side of control fabric and ∼42°C above ambient air. Conversely, the temperature of DRF in cooling mode is ∼5°C below the white side and ∼13°C above the air (Figure S18).

To further evaluate the actuation and recovery performance of HYAM rotating a fabric like a “spindle” driving a “gate”, a HYAM is sewn 1 mm from the edge of one side of the DRF. For practical applications, we test the performance by mimicking the humidity changes around the fabric, considering the daily environmental humidity fluctuations and the microenvironment between human skin and clothing caused by sweat evaporation. Figure 5B displays the time dependence for DRF (∼29 mg) driven by a 1.01 mg HYAM (2 strands, 3000 tpm) to switch between warming and cooling modes for one sorption–desorption cycle. Herein, the DRF is in one mode when the environment humidity is 30–50%, then with the humidity increasing to 70–90%, it gradually rotates until reaching 180° at approximately 9 s. When the humidity decreases and a voltage of 15 V is supplied, DRF swiftly rotates back and returns to its initial mode within 4.6 s. During 100 sorption–desorption cycles, warming and cooling mode switching for DRF performs extremely stably (Figure 5C).

The twist direction of HYAM and its relative position with DRF can be determined according to the specific application, to regulate mode switching on demand. For the case of adaptive curtains, the DRF cools the indoors on hot and dry midday; with the humidity increasing and the temperature dropping, the HYAM is actuated and induces the curtains to flip, keeping people in the building warm (Figure 5D and Movie S3). The curtain pattern was designed for a model house, and the field test was conducted in Kowloon, Hong Kong (October 12–13, 2024). We also construct pristine fabrics with the white side and the black side for comparison. The experimental setup is shown in Figure 5E, with walls and roofs other than the test one coated with white paint to reduce their effects on the indoor temperature. A thermocouple is placed in the middle of the house to record the indoor air temperature. The test results are displayed in Figure 5D‐iii and iv and Figure 5F. After sunrise, the indoor air temperature increases rapidly with solar intensity. The indoor temperature of the house equipped with smart curtains in cooling mode is always lower than that of white fabric during the daytime, confirming the radiative cooling effect of DRF, which can also be proved by the thermal images (top of Figure S20). The DRF provides the largest temperature reduction of 3.4°C compared to white fabric and 8.3°C regarding to the ambient air, with a net cooling power of ∼107.3 W/m^2^. The integration of HYAM is expected to deliver effective cooling performance with controllable actuation strategy and reasonable power input. After the sun sets, the indoor temperature drops, and the humidity gradually rises. Then, the DRF switches to the warming mode. A temperature increase can be detected in the modal house with DRF after switching the mode (Figure 5D‐iii). However, considering the reduction of MIR emission from the human body did not actually occur in the mould house, the indoor air temperature between the houses with DRF and black fabric is similar (bottom of Figure S20). The next day, to switch to the cooling mode, the DRF can be actuated by the humidity decrease due to sunrise, or controllably adjusted by electrothermal actuation, thereby cooling the indoors as desired (Figure 5D‐iv).

To explore more potential applications of reversible HYAM in adaptive thermal management, we fabricate a smart clothing based on a human sweating map [37] and evaluated its performance in dynamically changing humidity environments. As shown in Figure S21, the adaptive clothing is placed in an environment of ∼70% humidity. To mimic the increased humidity in the microenvironment under clothing caused by sweat evaporation from a hot human, the humidity is raised to ∼85%. Each HYAM (∼2.39 mg) successfully actuates the DRF (∼60.5 mg), which weighs over 25 times its weight, to the cooling mode within ∼64 s. When the humidity decreases and a voltage of 10 V is applied, the DRF quickly returns to the warming mode in just ∼27 s (Movie S4). Noteworthy is that the surface temperature of HYAM is ∼38°C, which is lower than the skin burn temperature of 44°C [38], confirming the application safety in wearables. These results demonstrate that HYAM holds great promise for regulating human thermal comfort under various individual conditions.

## 3 Conclusion

Inspired by the autonomous movement of skeletal muscles, we have developed HYAM, a novel artificial muscle designed for adaptive thermal management. This innovative HYAM combines moisture‐responsive actuation with controllable electrothermal recovery, achieving rapid, precise, and reversible movements. Compared to traditional artificial muscles, HYAM improves actuation stroke and speed by 128% and 136%, respectively, while significantly shortening recovery and full cycle times by 91% and 83%. Geometric characterization and finite element simulations reveal that HYAM's actuation is driven by fiber volume changes caused by moisture absorption and desorption. Its fast and reversible actuation enables seamless integration with DRFs for adaptive and switchable thermal management. For instance, HYAM‐driven adaptive curtains can switch to a cooling mode, reducing indoor temperatures by 3.4°C compared to standard fabrics, or transition to a warming mode as humidity increases, providing warmth to occupants.

With superior actuation performance, recovery efficiency, and mechanical properties, HYAM demonstrates all‐around excellence among yarn‐based artificial muscles. Furthermore, HYAM offers promising applications in intelligent clothing for personal thermoregulation, extending the use of artificial muscles to adaptive thermal management. Future work will focus on enhancing HYAM's long‐term stability under complex environmental conditions and optimizing fabrication for scalability and cost‐efficiency. Applications in areas such as intelligent clothing, energy‐efficient building materials, and soft robotics hold great promise, paving the way for broader adoption of HYAM in smart textile innovations.

## 4 Materials and Methods

### 4.1 Preparation of PYAM, SHYAM, and HYAM

Pristine viscose fiber was purchased from Bo La Chemical Fiber Co., Ltd, Shaoxing, China. This fiber has a section width of ∼108 µm and a count of 10 denier (equivalent to a diameter of ∼58 µm). The PYAM was fabricated by first fixing the viscose fiber with clamps at both ends of the twisting machine, and the twist was inserted by rotating the motor at one end. At the other end, a movable clamp allowed for free adjustment of fiber shortening due to twist insertion. This bench‐scale twisting machine enables the automated and scalable fabrication of twisted fibers. Afterward, the twisted fiber was then folded in its middle by attaching a load of 70 mg, thereby plying its two sections together to form a PYAM. The preparation process of SHYAM and HYAM was the same as the above one, with one difference before twisting. Before the twisting process for SHYAM, half of the viscose fiber was coated with Ti_3_C_2_T_x_ MXene flakes to form a sheath, by drawing it from a gel‐like state MXene droplet along the length of the vertically suspended fiber. The MXene dispersion (∼0.1–0.5 or ∼0.5–5 µm in diameter, ∼100 mg/ml in concentration) was from Xinxi Technology Co., Ltd, Foshan, China. During the folding and self‐plying, the load was attached at the boundary between the all‐in‐one pristine and coated fiber. In terms of the HYAM, the whole fiber was drawn from this MXene droplet. Depending on the desired conductivity and torsional actuation, this droplet‐coating method was repeated multiple times. The overcoating MXene flakes were squeezed out during the twisting process due to the tighter fiber structure.

### 4.2 Fabrication of DRF

A DRF based on a double‐sided design is adopted (Figure S14), a white cotton woven fabric coated with PDMS and TiO_2_ nanoparticles, and a black cotton non‐woven liner coated with MXene flakes. Before all the processing, the woven fabric was ultrasonicated with ethanol/acetone solution with a 1:1 volume ratio for half an hour, then dried in the air to obtain a stable fabric shape and remove grease from its surface. The solution with TiO_2_ nanoparticles was prepared as follows. First, 8 g TiO_2_ nanoparticles with a diameter of 100 nm (Aladdin) were dispersed into 200 mL tetrahydrofuran (THF). This solution was ultrasonicated with 120 W for 30 min, then magnetically stirred at room temperature for another 30 min, to achieve homogenous dispersion. Afterward, it was added with 8 g PDMS A and ultrasonicated for 30 min. Another solution was prepared by adding 0.8 g PDMS B into 200 mL THF, then magnetically stirring for 30 min. Finally, the above two solutions were mixed and magnetically stirred for 12 h, and the coating solution was completed. The washed woven fabric was immersed in the coating solution for 5 min. Then, it was transferred to a fume hood for 15 min to allow THF to evaporate, followed by placing it in an oven at 80°C for 10 min. This coating process was repeated 6 times to achieve a good coating rate of PDMS and TiO_2_ nanoparticles. The black side was seamlessly laminated with this coated fabric by hot pressing. Hereafter, the MXene dispersion (∼0.5–5 µm in diameter, ∼60 mg/ml in concentration) from Xinxi Technology Co., Ltd, was coated on the black surface of this laminated fabric. Ultimately, the DRF was fabricated with a black side coating of MXene flakes for warming mode and a white side coating of PDMS and TiO_2_ for cooling mode.

### 4.3 Moisture Actuation and Natural/Electrothermal Recovery Analysis

Two self‐made acrylic boxes (dimensions of 35 cm in length, 35 cm in width, and 75 cm in height) were used as the testing chambers for wetting and drying, where a humidifier and a dehumidifier were placed, respectively. Before the actuation, the yarn‐based muscles were freely hung in the drying chamber for at least 5 min, with the dehumidifier creating an environment of 30–50% RH to balance the muscle torque at a low humidity condition. After that, they were quickly transferred to the wetting chamber with the humidifier on (water fog spraying speed of 0.1 g/s) for 10 s actuation. The humidifier was then turned off, and the muscles were immediately transferred to the drying chamber for natural recovery or stayed in place with a direct voltage supplied for electrothermal recovery. Two poles of the power supply were connected with the top and bottom ends of the yarn‐based muscles by silver‐coated nylon wires (20D/12F, ∼50Ω/cm, Lanxin New Material Weaving Technology Co., Ltd., Dongguan, China). The cyclic response of muscles to moisture was conducted through this repeated humidification‐dehumidification process. The whole process was captured by the Sony A7M2 and analyzed frame‐by‐frame via tracker.

### 4.4 Radiation Regulation Measurement

The spectral reflectivity in the ultraviolet, visible, and near‐infrared wavelength ranges was determined using a UV–vis–NIR spectrometer (Lambda 950, PerkinElmer) equipped with an integrating sphere. The IR reflectivity (ρ) and transmittance (τ) were measured using an FTIR spectrometer (Spotlight 200i, PerkinElmer) accompanied by an infrared integrating sphere. The IR emissivity (ε) was then calculated using the equation ε = 1 − ρ – τ. To explore the cooling and heating performance outdoors, the daytime measurement was conducted on hot sunny days in Kowloon, Hong Kong (14.18° E, 22.30° N) using the customized experimental devices (Figure S17), with real‐time solar irradiance tracked by pyranometer (TES132, TES Electrical Electronic), temperature recorded by thermocouples (APPPLENT, AT4208) and an infrared thermal imager (Fluke TIS65, USA). The thermocouples were covered by fabrics, and a thermocouple was exposed to the air without direct sunlight to detect the ambient temperature variations.

### 4.5 Statistical Analysis

All experiments were tested three times (n = 3). The reported values with scale bars were calculated as mean and standard deviation, and the curves were generated by polynomial fitting. Statistical analysis of the data was plotted using Origin 2021.

## Conflicts of Interest

The authors declare no conflict of interest.

## Supporting information




**Supporting File 1**: advs73704‐sup‐0001‐SuppMat.pdf.

**Supporting File 2**: advs73704‐sup‐0002‐MovieS1.mp4.

**Supporting File 3**: advs73704‐sup‐0003‐MovieS2.mp4.

**Supporting File 4**: advs73704‐sup‐0004‐MovieS3.mp4.

**Supporting File 5**: advs73704‐sup‐0005‐MovieS4.mp4

## Data Availability

The data that support the findings of this study are available from the corresponding author upon reasonable request.
