# Supplementary material for: Dual‐Responsive Yarn‐Based Artificial Muscles for Adaptive Thermal Management
Source: Adv Sci (Weinh). 2026 Jan 9;13(17):e21717. doi: 10.1002/advs.202521717 (PMC13042714; doi:10.1002/advs.202521717)
Supplement: Supplementary file 1 — Supporting File 1: advs73704‐sup‐0001‐SuppMat.pdf. [file ADVS-13-e21717-s001.docx]

Supplementary Materials for

**Dual-responsive yarn-based artificial muscles for adaptive thermal management**

Mengjiao Pan^1,2,3^, Xiaohui Zhang^1,2,3^, Jinhao Xu^1,2,3^, Ziqi Li^1,2,3^, Junjie Wang^1,2,3^, Qirui Zhang^1,2,3^, Yi Liu^1,2,3^, Hongfei Yang^1,2,3^, Kinor Jiang^2,3^, Albert Chan^2,4^, Yucan Peng^5*^, Dahua Shou^1,2,3,6*^

^1^ Future Intelligent Wear Centre, School of Fashion and Textiles, The Hong Kong Polytechnic University, Kowloon 999077, Hong Kong, People’s Republic of China.

^2^ Research Centre of Textiles for Future Fashion, The Hong Kong Polytechnic University, Hung Hom, Kowloon 999077, Hong Kong, People’s Republic of China.

^3^ Research Institute for Intelligent Wearable Systems, The Hong Kong Polytechnic University, Hung Hom, Kowloon 999077, Hong Kong, People’s Republic of China.

^4^ Department of Building and Real Estate, The Hong Kong Polytechnic University, Hung Hom, Kowloon 999077, Hong Kong, People’s Republic of China.

^5^ Department of Energy and Resources Engineering, College of Engineering, Peking University, Beijing 100871, China.

^6^ PolyU-Xingguo Technology and Innovation Research Institute, The Hong Kong Polytechnic University, Hung Hom, Kowloon 999077, Hong Kong, People’s Republic of China.

* Corresponding authors:

Email: [dahua.shou@polyu.edu.hk](mailto:dahua.shou@polyu.edu.hk) (D. Shou); [yucan.peng@pku.edu.cn](mailto:yucan.peng@pku.edu.cn) (Y. Peng)

**This PDF file includes:**

Supplementary Text

Figs. S1 to S21

Tables S1 to S2

Movies S1 to S4

Supplementary Text

Simulation details

The simulation is conducted based on the following consumptions:

1. The twisting trajectory of the fibers is the equal helix.

2. Each fiber is identical in nature, whose cross section is circular and uniform along its length.

3. The radial pressure on the yarn axis caused by the fiber torsion is equal everywhere along its entire length.

The geometric model and simulations are conducted using the Abaqus program. The diameter of the fiber is 58 μm, and the length of the simulation unit is 2 mm. Yarn muscle has a twist density of 1000 turns per m. The fiber density and elasticity are specified. The hygroscopic swelling behavior is modeled by defining orthotropic hygroscopic expansion coefficients, which generate the driving force for torsion. A dynamic explicit analysis step is then employed. By applying appropriate boundary conditions to the yarn, only one end is allowed to twist, while the motion of the other rigid bodies is restricted. Moisture absorption is simulated using the thermal analogy, where the temperature variable in a predefined field is defined to represent moisture concentration. An increase in this concentration from an initial value to a target saturation value simulates the transition from a dry to a wet state.


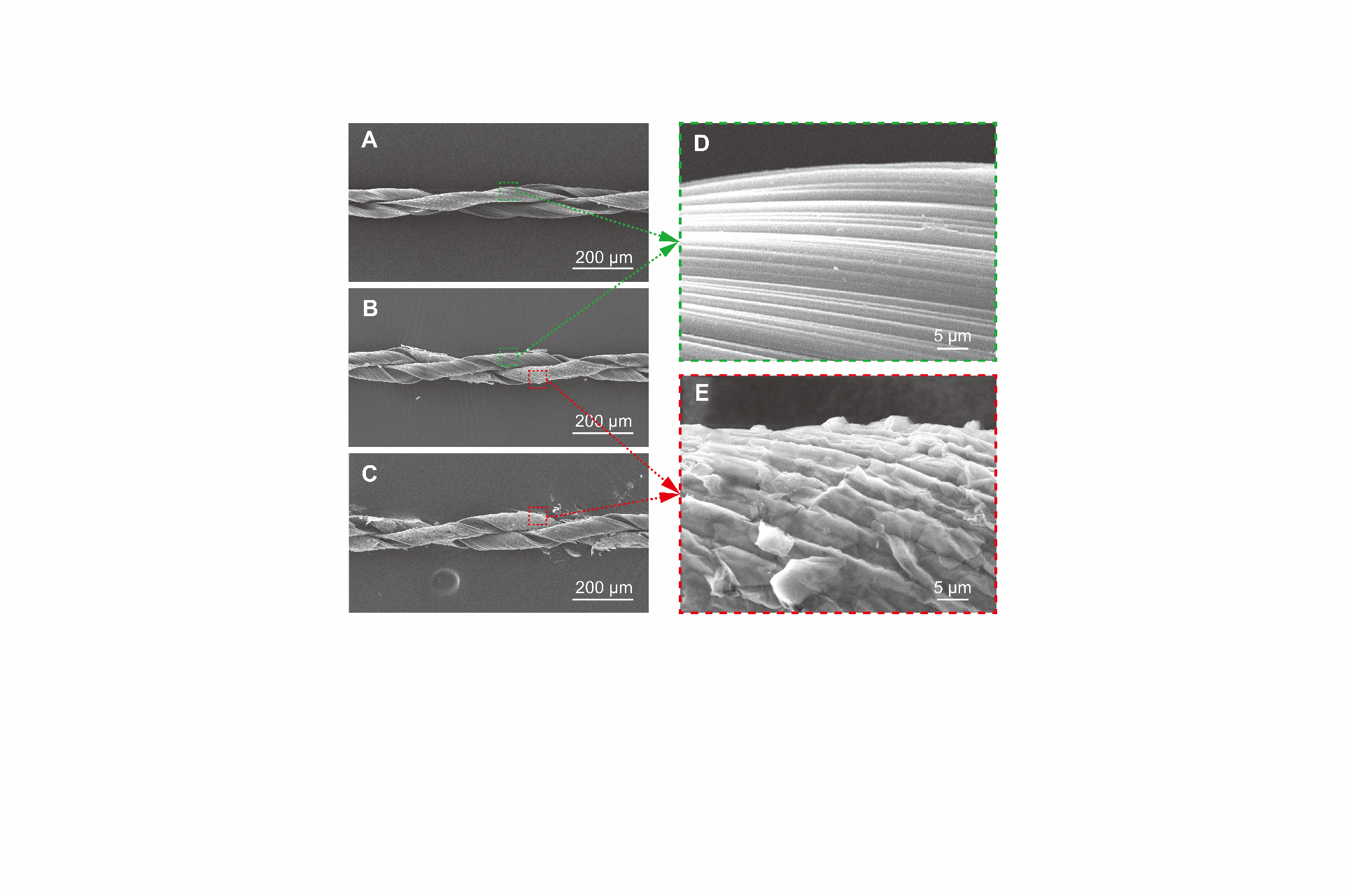


Fig. S1. Low magnification SEM images of (A) PYAM, (B) SHYAM, and (C) HYAM. High magnification SEM images of the (D) pristine and (E) 100 mg/ml MXene-coated fiber surface.


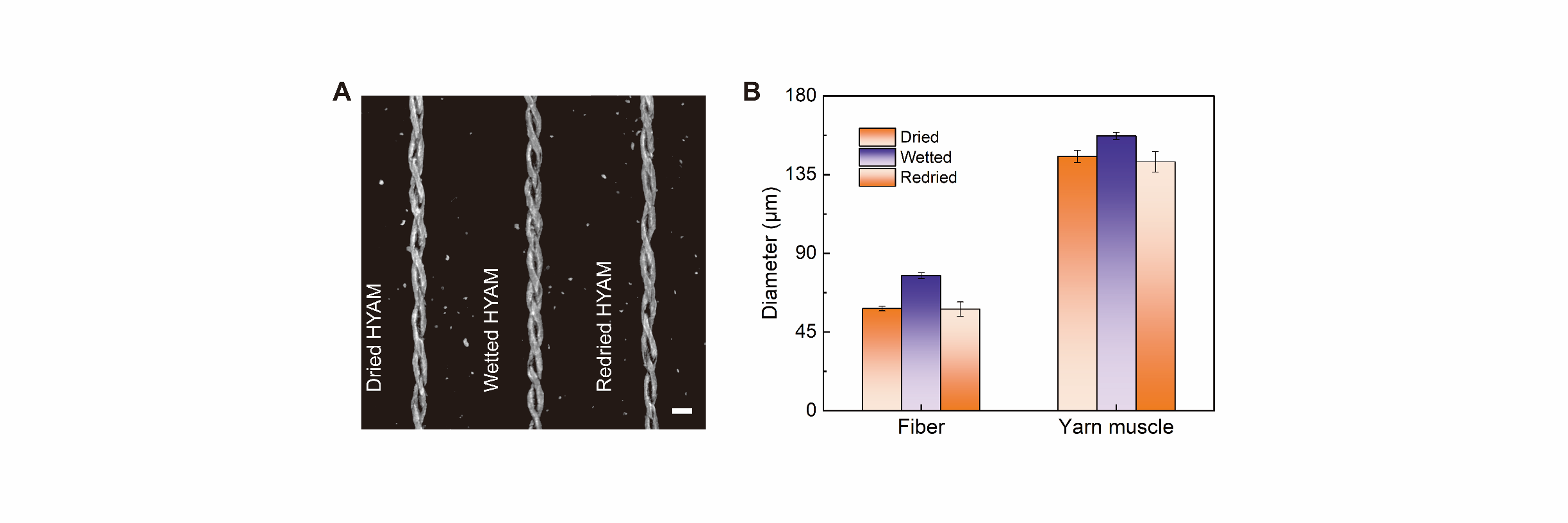


Fig. S2. Diameter characterizations of HYAM. (A) Optical images of dried HYAM, wetted HYAM, and redried HYAM, respectively (scale bars = 200 µm). (B) Diameter comparison of the single fiber and yarn muscle among dried HYAM (58 and 145 µm), wetted HYAM (77 and 157 µm), and redried HYAM (58 and 142 µm).


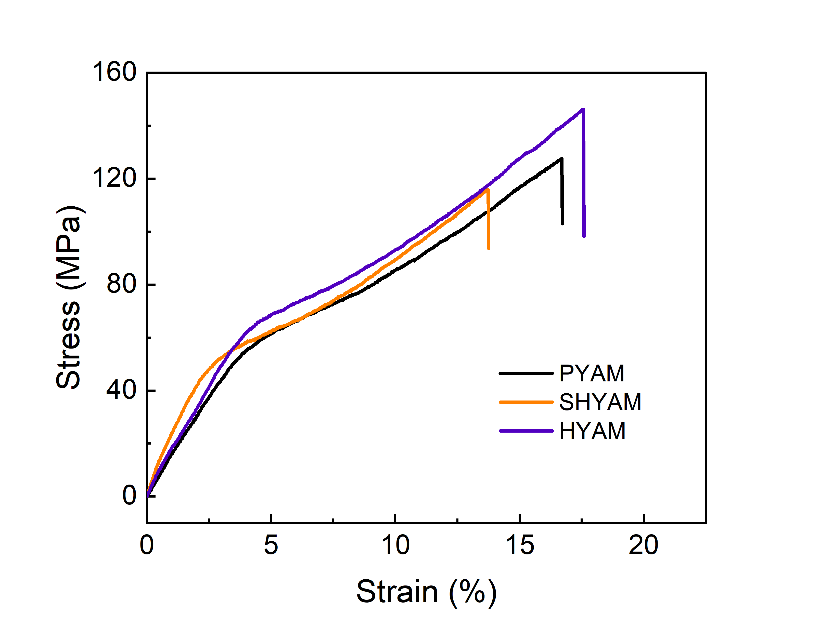


Fig. S3. Comparison of tress-strain curves among PYAM, SHYAM, and HYAM. The experiments are conducted under ambient conditions at ~30%RH and 25°C.


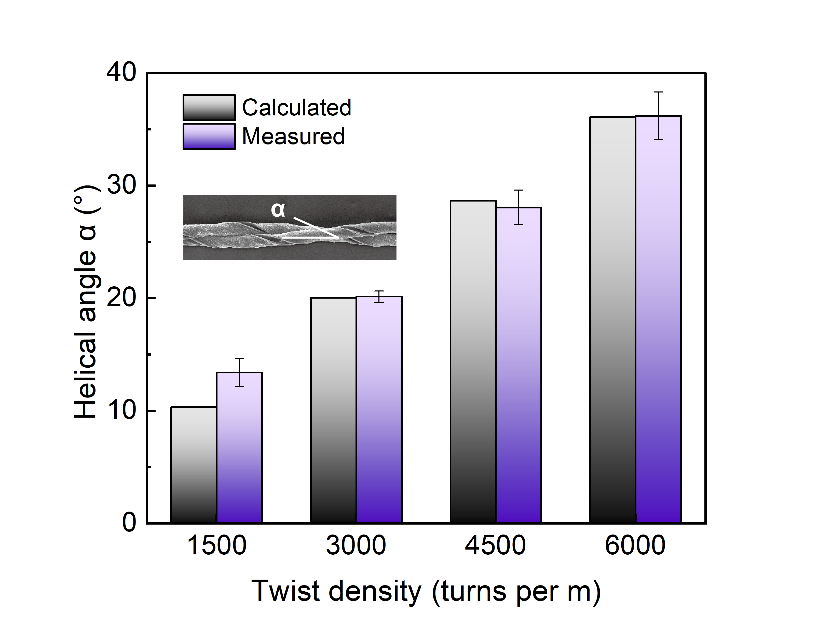


Fig. S4. Dependence of helical angle (α) on the inserted twist for a one-strand self-balanced HYAM.


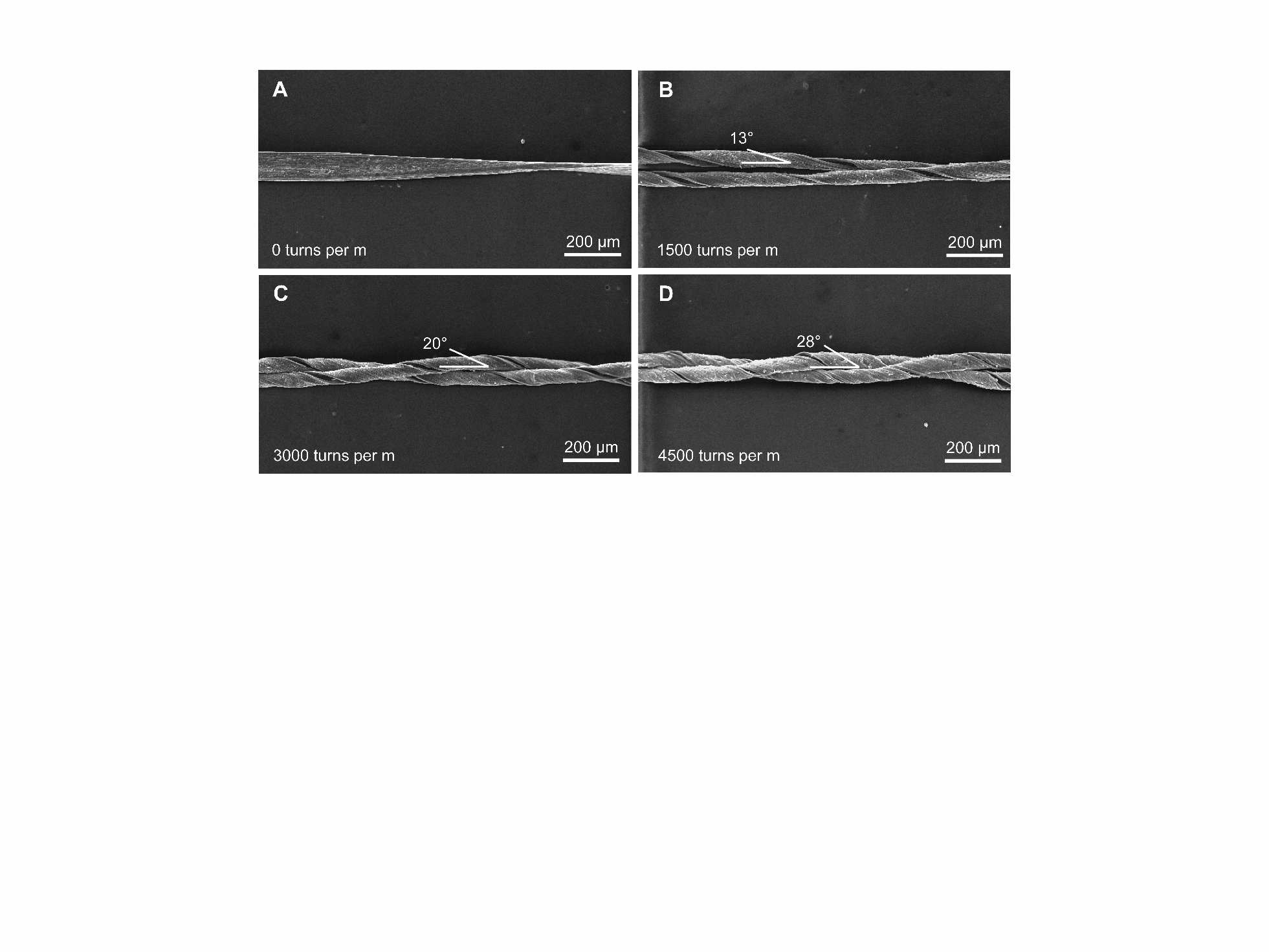


Fig. S5. SEM images of one-strand HYAM with twist densities of (A) 0, (B) 1500, (C) 3000, and (D) 4500 turns per meter, respectively.


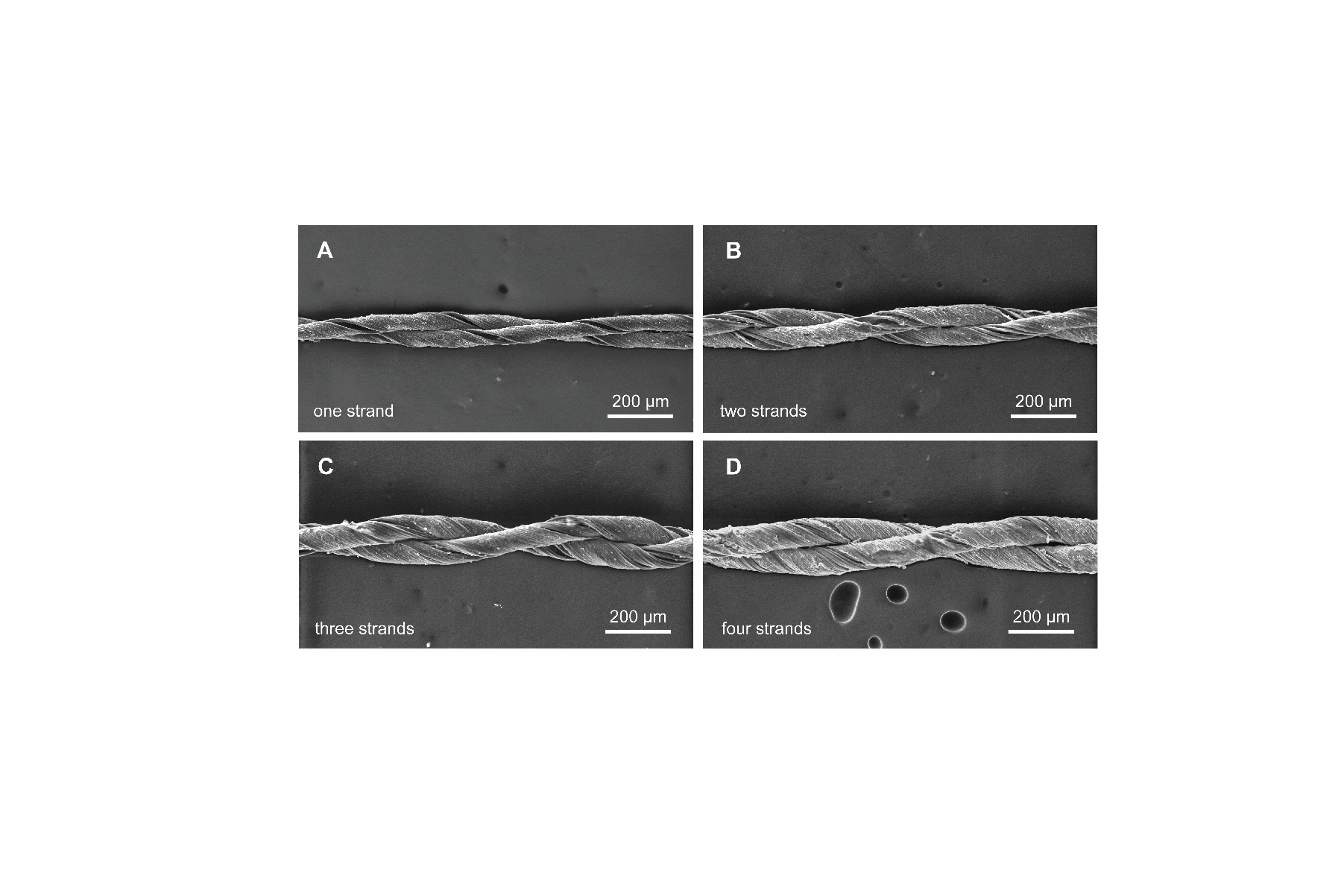


Fig. S6. SEM images of HYAM with (A) one strand, (B) two strands, (C) three strands, and (D) four strands, respectively. Each HYAM has a twist density of 3000 tpm.


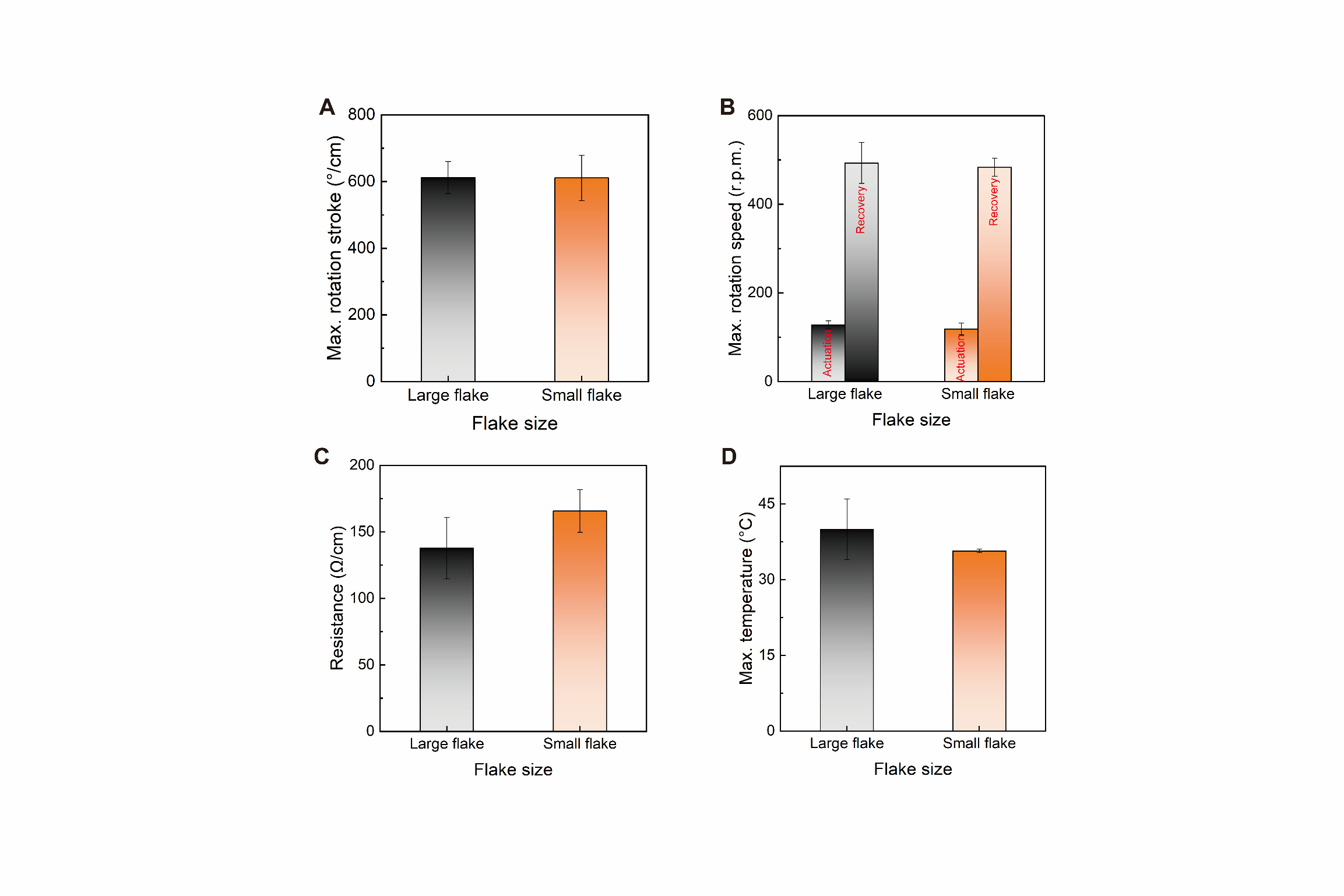


Fig. S7. Effect of MXene flake size. Comparison of (A) Max. rotation stroke, (B) Max. rotation speed, (C) Resistance, and (D) Max. heated temperature between HYAM coated with MXene (~100 mg/ml) of large (0.5-5 μm in diameter) and small (0.1-0.5 μm in diameter) flakes once. Each HYAM is formed by two strands of fiber with a twist density of 3000 tpm.


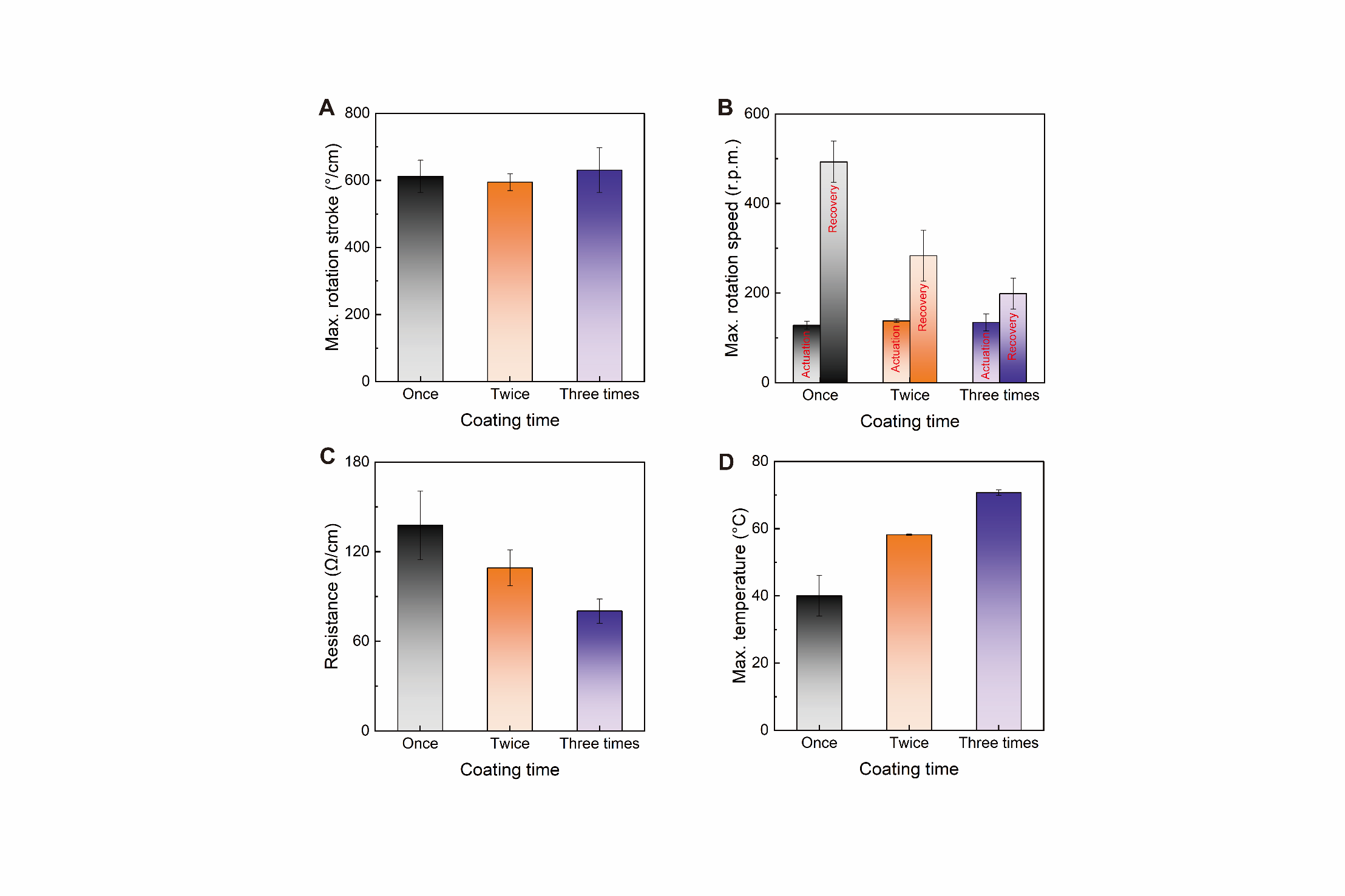


Fig. S8. Effect of MXene coating time. Comparison of (A) Max. rotation stroke, (B) Max. rotation speed, (C) Resistance, and (D) Max. heated temperature among HYAM coated with MXene once, twice, and three times. Each HYAM is formed by two strands of fiber with a twist density of 3000 tpm. The coating is MXene with large flakes.


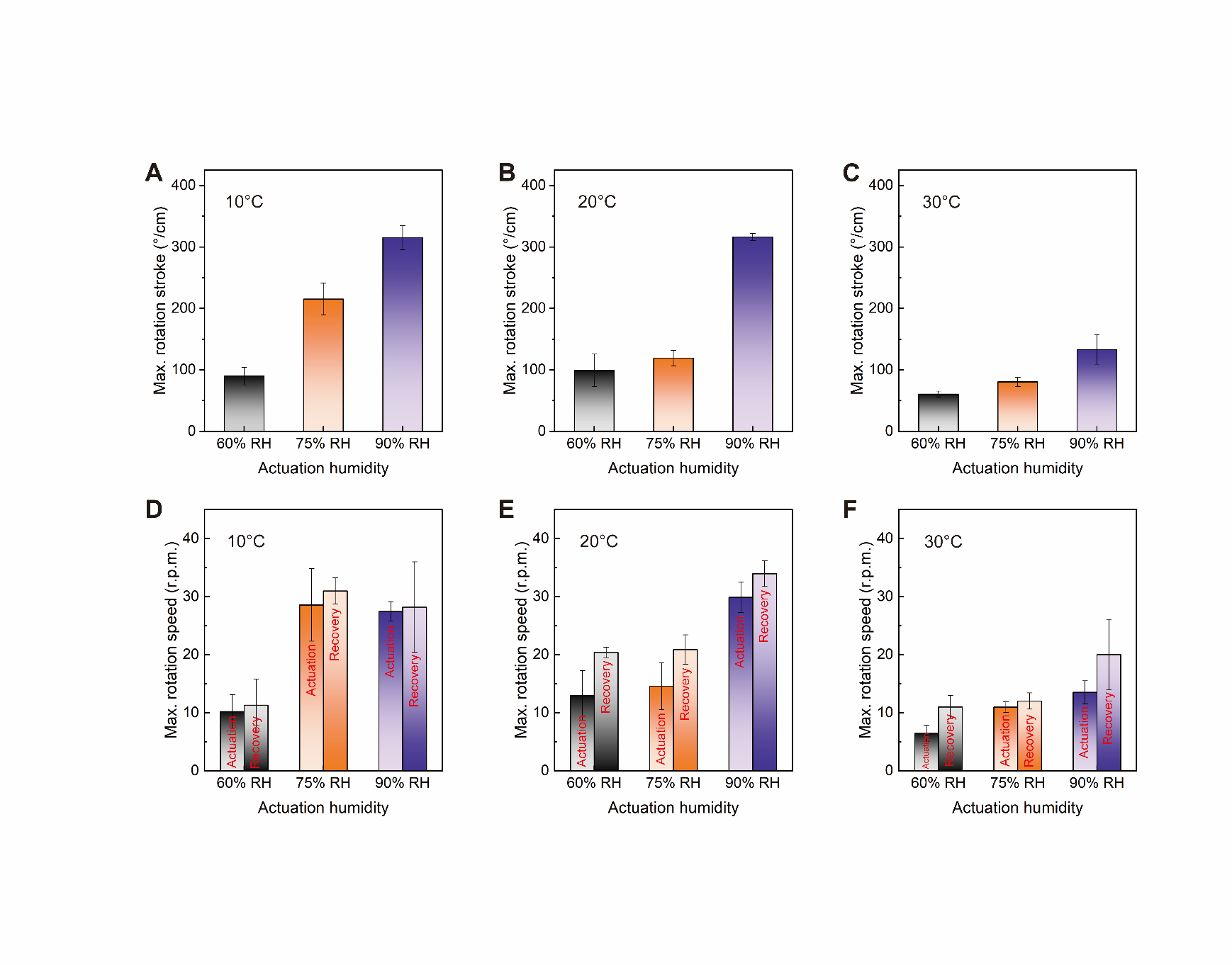


Fig. S9. Effect of ambient temperature and actuation humidity. (A, B, and C) Effect of different actuation humidities on the Max. rotation stroke at different ambient temperatures (A 10°C, B 20°C, C 30°C). (D, E, and F) Effect of different actuation humidities on the Max. rotation speed at different ambient temperatures (A 10°C, B 20°C, C 30°C). Each HYAM is formed by two strands of fiber with a twist density of 3000 tpm, and is coated with large MXene flakes once. The HYAM is actuated from 45% ± 2% RH to 60%±5% RH, 75% ± 5% RH, and 90% ± 5% RH for 30 seconds.

**
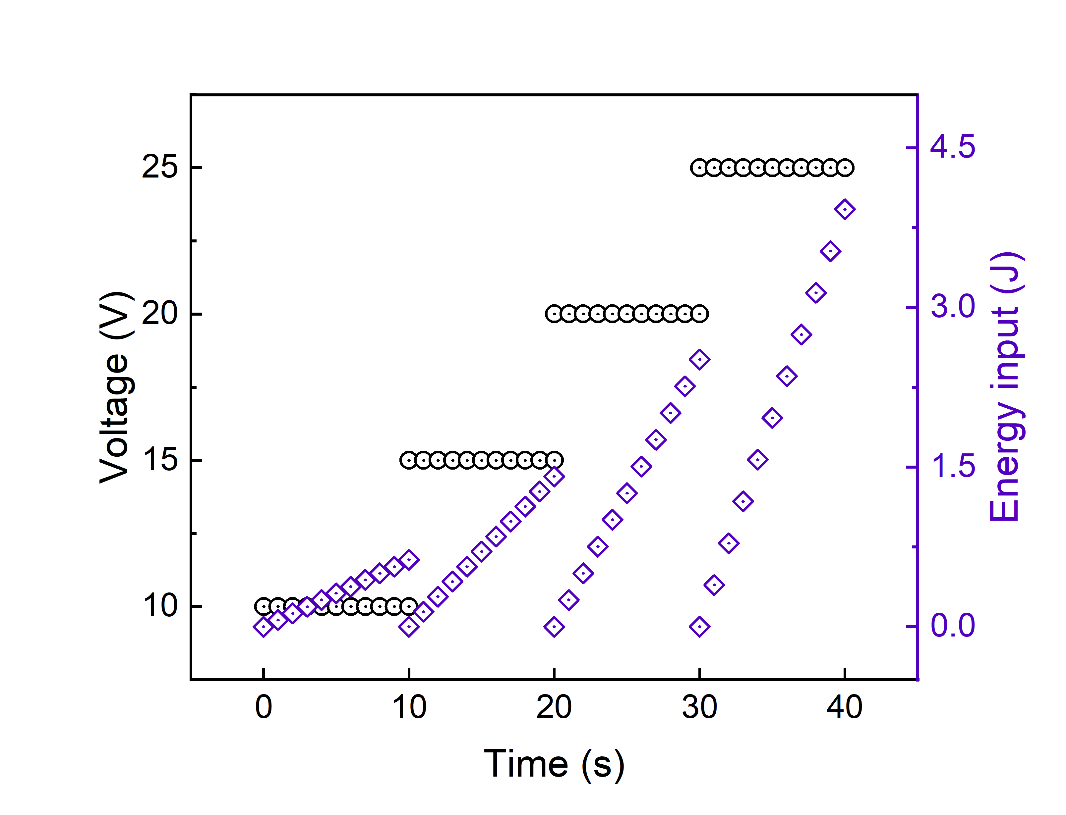
**

**Fig. S10. The energy consumption of HYAM under different supplied voltages for 10-second electrothermal drying per cycle**. The HYAM is formed by two strands of fiber with a twist density of 3000 tpm and coated with large MXene flakes once.


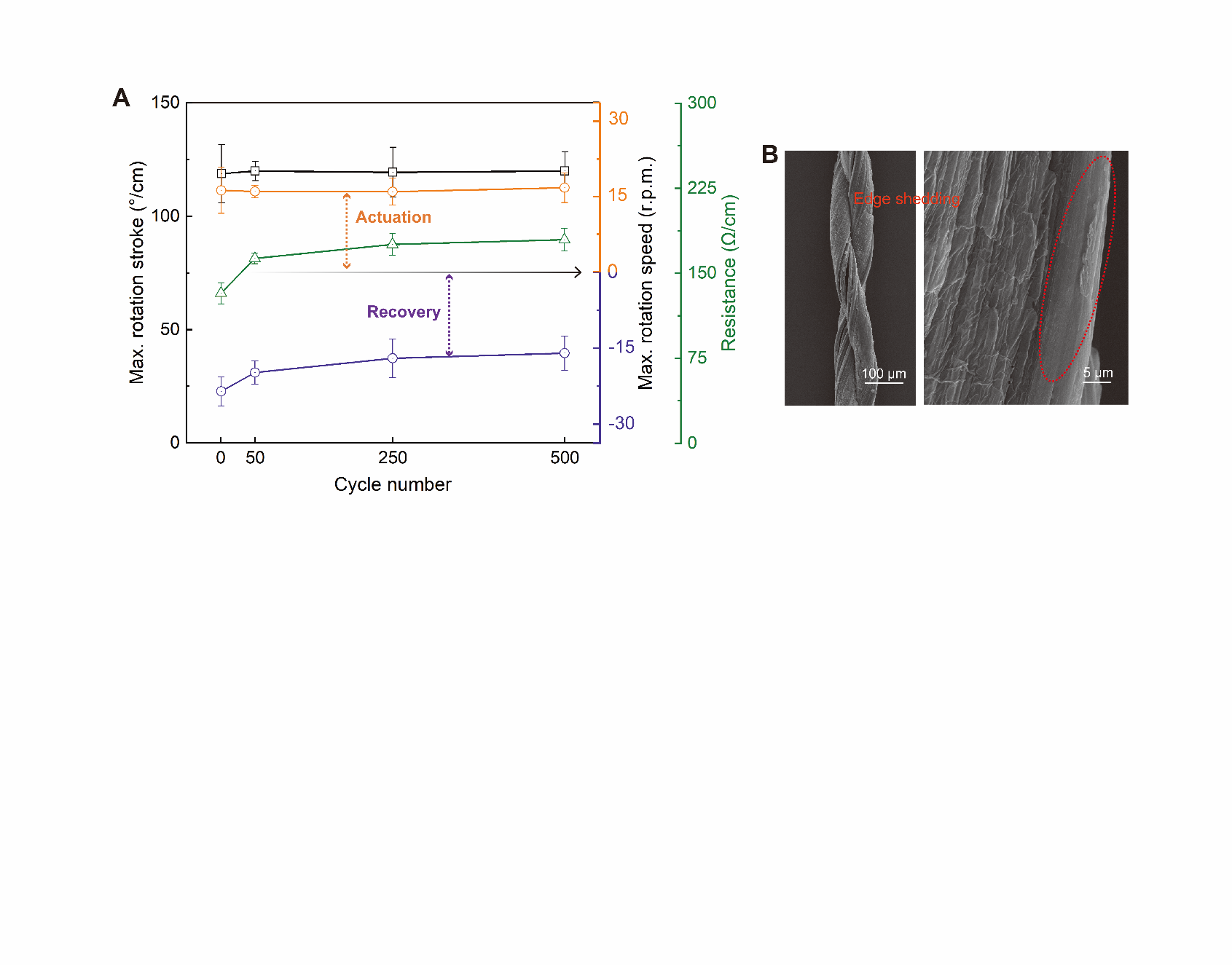


Fig. S11. Effect of long-time humidity actuation and electrothermal recovery. (A) Comparison of Max. rotation stroke and speed, and resistance of HYAM during 500 sorption-desorption cycles. Each HYAM is formed by two strands of fiber with a twist density of 3000 tpm, and is coated with large MXene flakes once. The HYAM is actuated from 45% ± 2% RH to 75% ± 5% RH at 20°C for 30 seconds. (B) The morphologies of HYAM after 500 cycles.


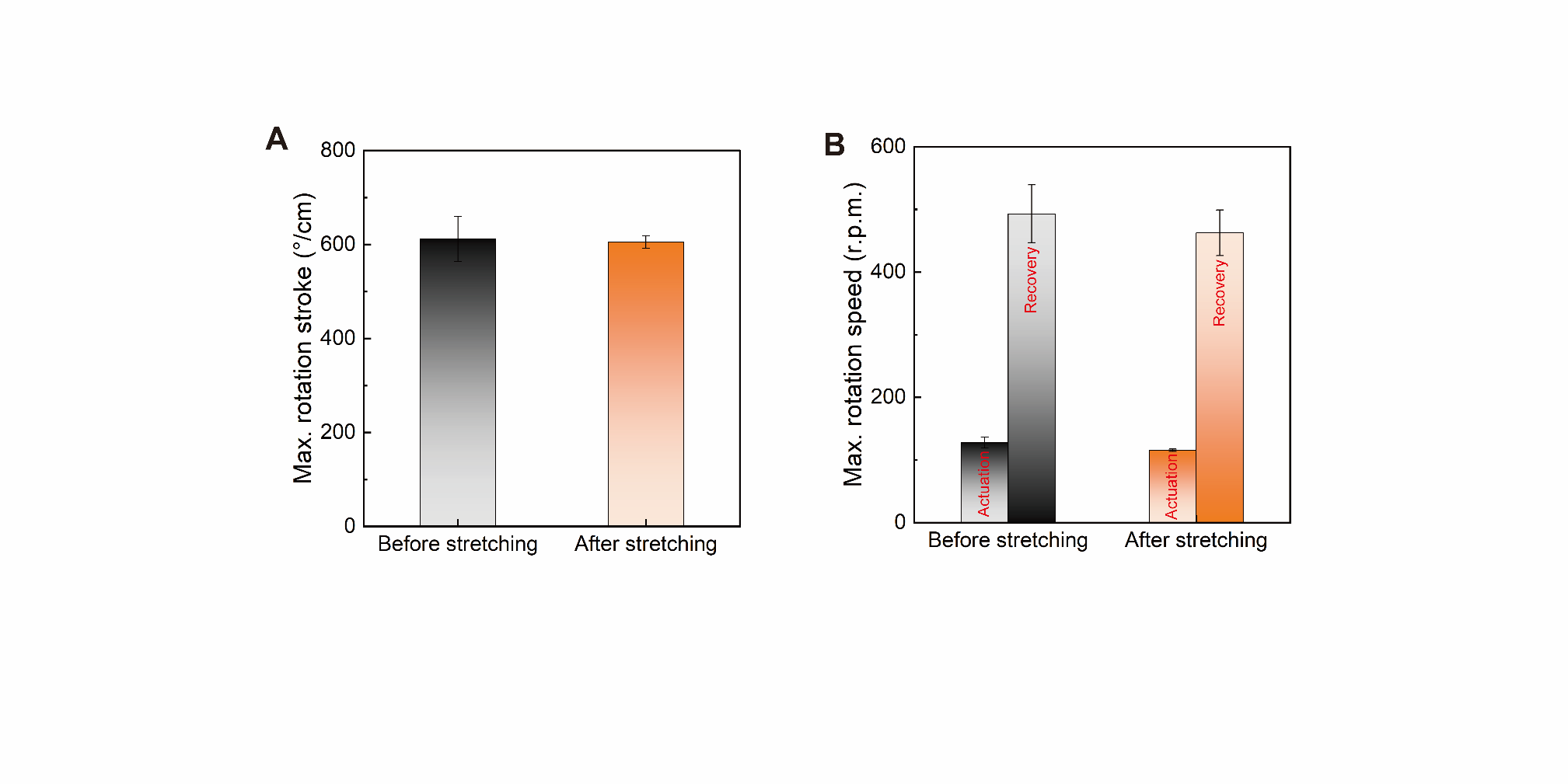


Fig. S12. Effect of long-time stretching. Comparison of (A) Max. rotation stroke, and (B) Max. rotation speed of HYAM before and after 10000 stretches. Each HYAM is formed by two strands of fiber with a twist density of 3000 tpm, and is coated with large MXene flakes once.

**
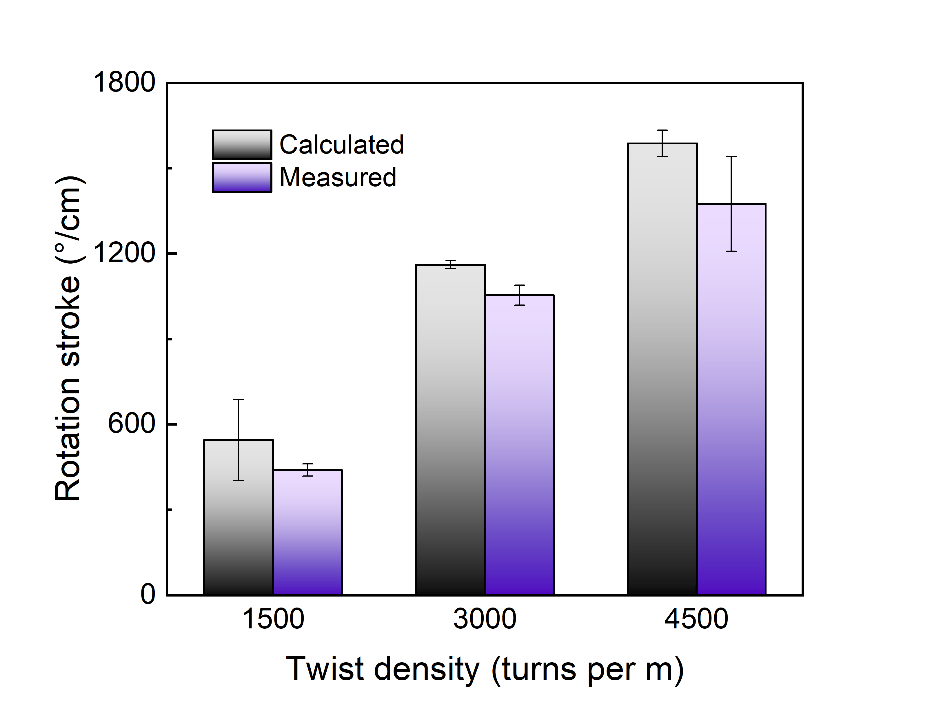
**

Fig. S13. Comparison of calculated and measured rotation stroke of HYAM with different twist densities. Each one-strand HYAM is coated with large-size MXene flakes once.


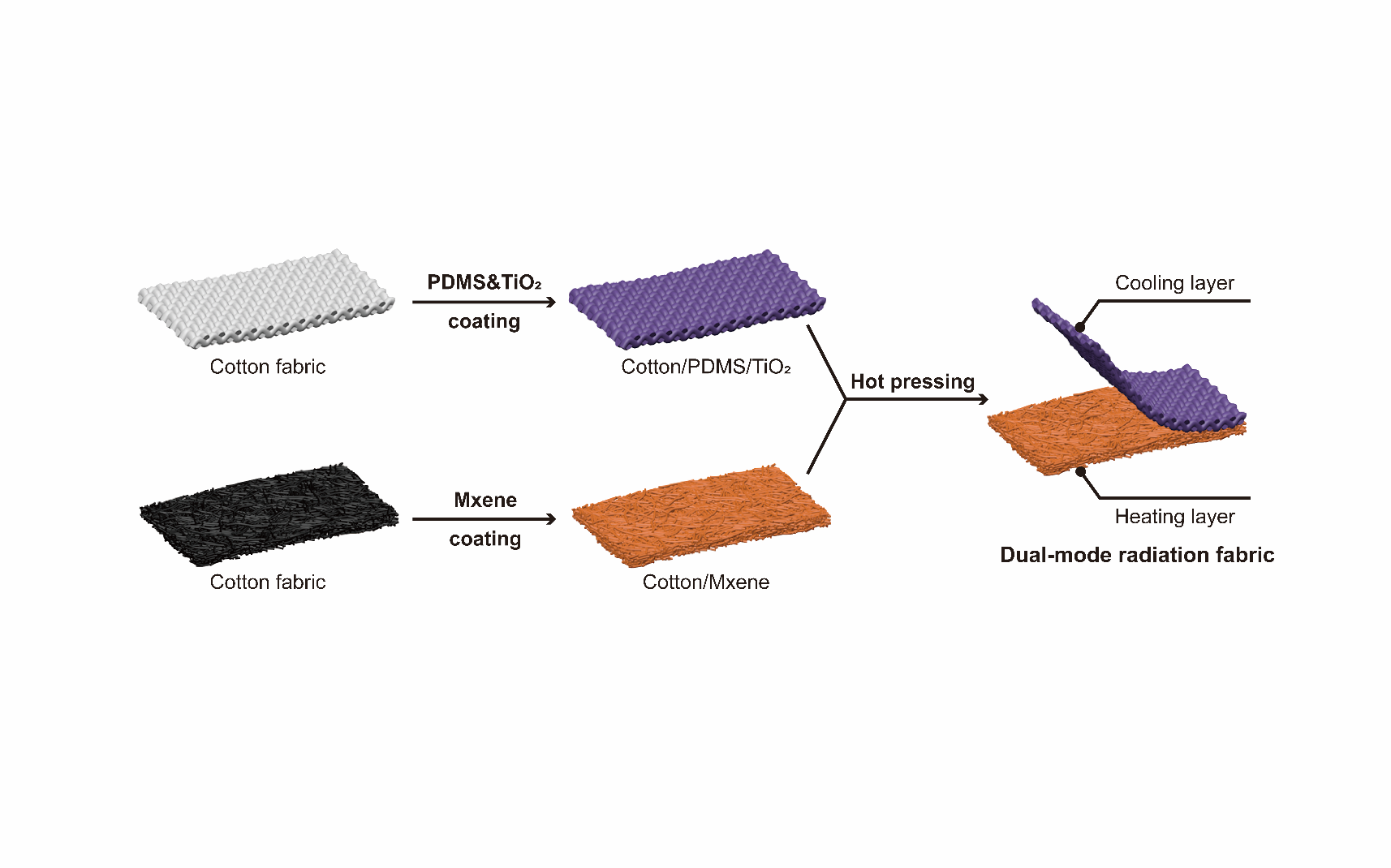


Fig. S14. Schematic illustration of the fabrication of a dual-mode radiation fabric.


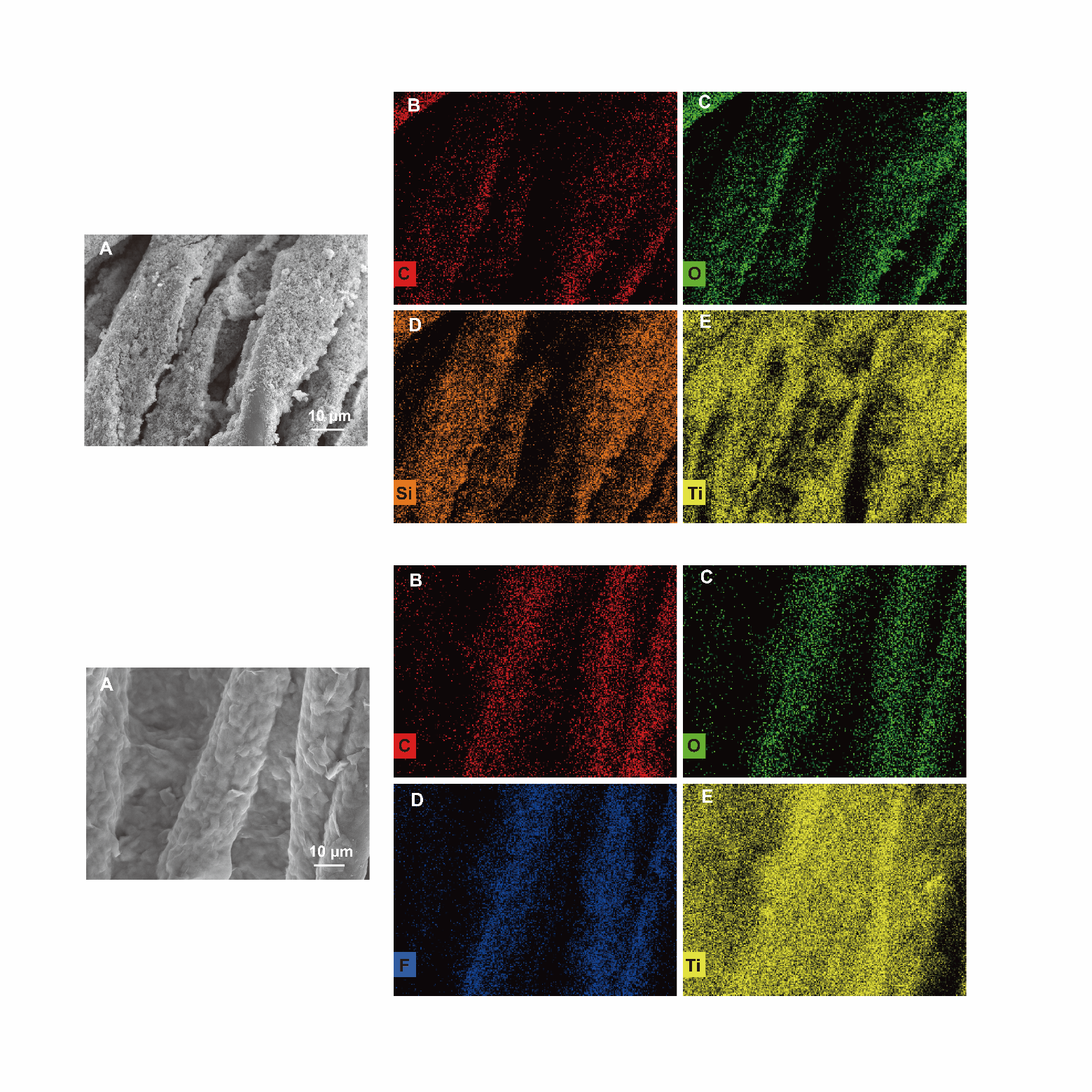


Fig. S15. SEM images (A) and EDX elemental mapping (B-E) of the cooling (top) and heating layer (bottom).


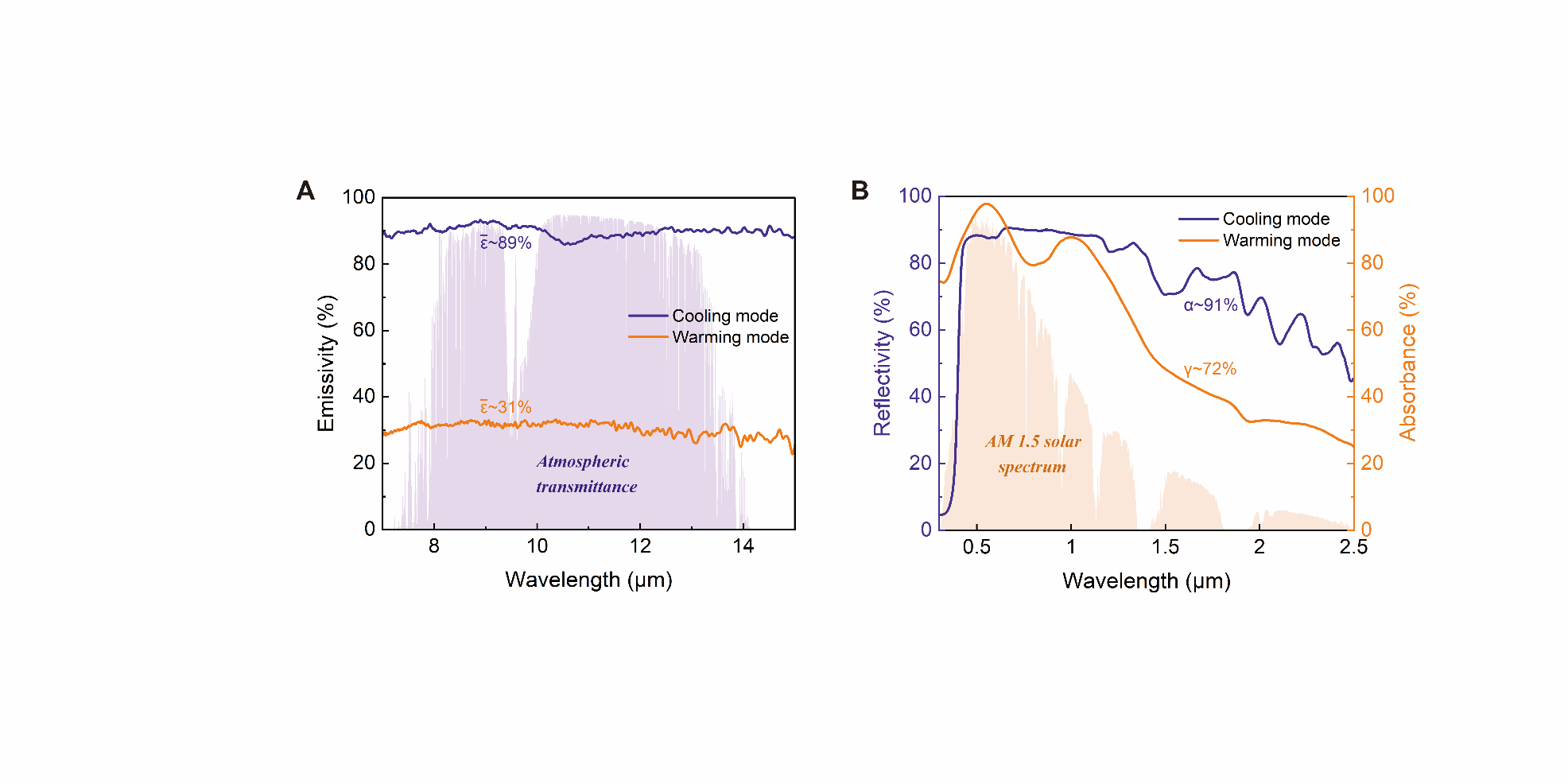


Fig. S16. The emissivity (A), reflectivity, and absorbance spectra (B) of DRF in cooling mode and warming mode.


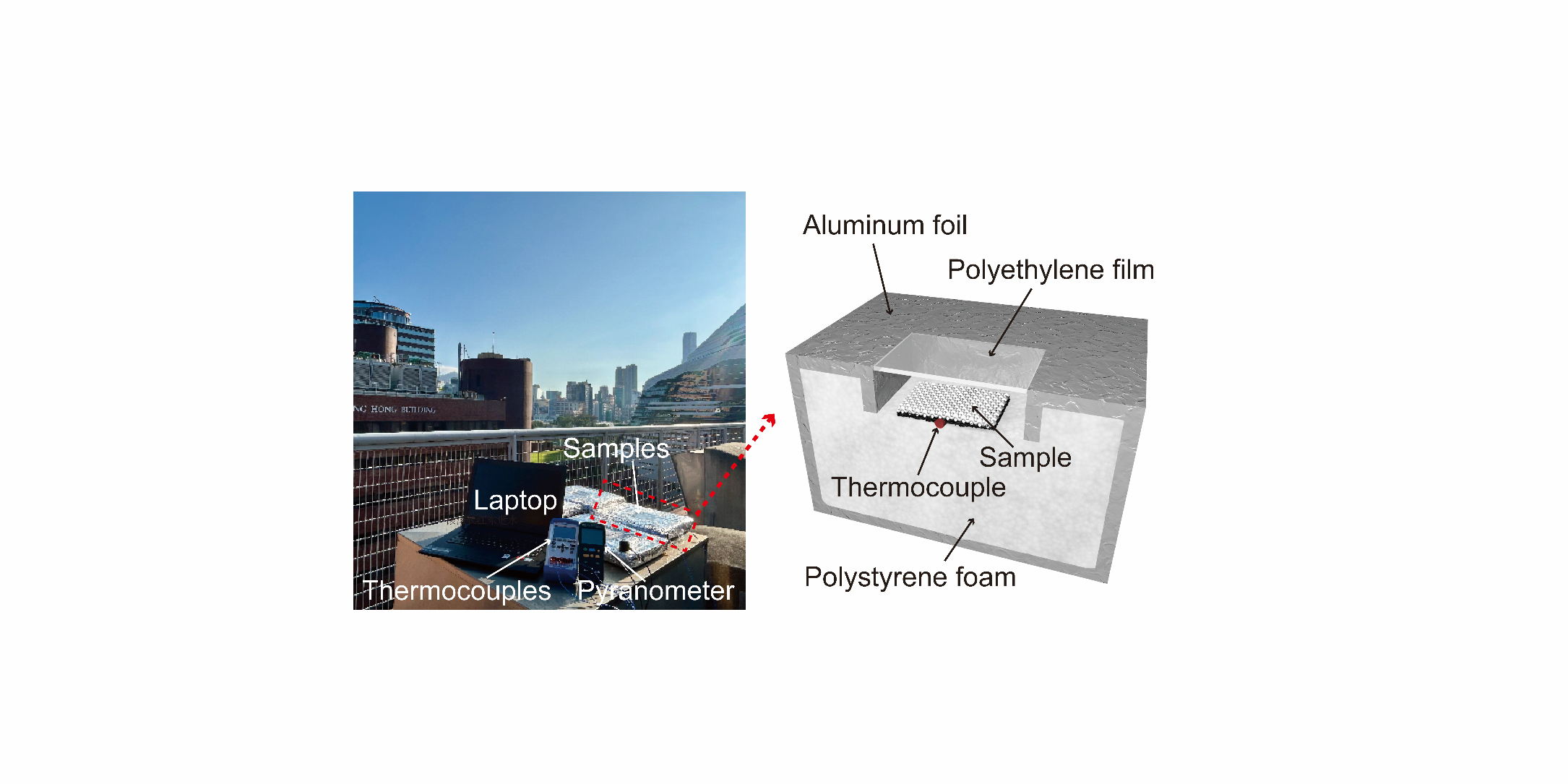


Fig. S17. Photograph and schematic illustration of the device for outdoor thermal measurements.


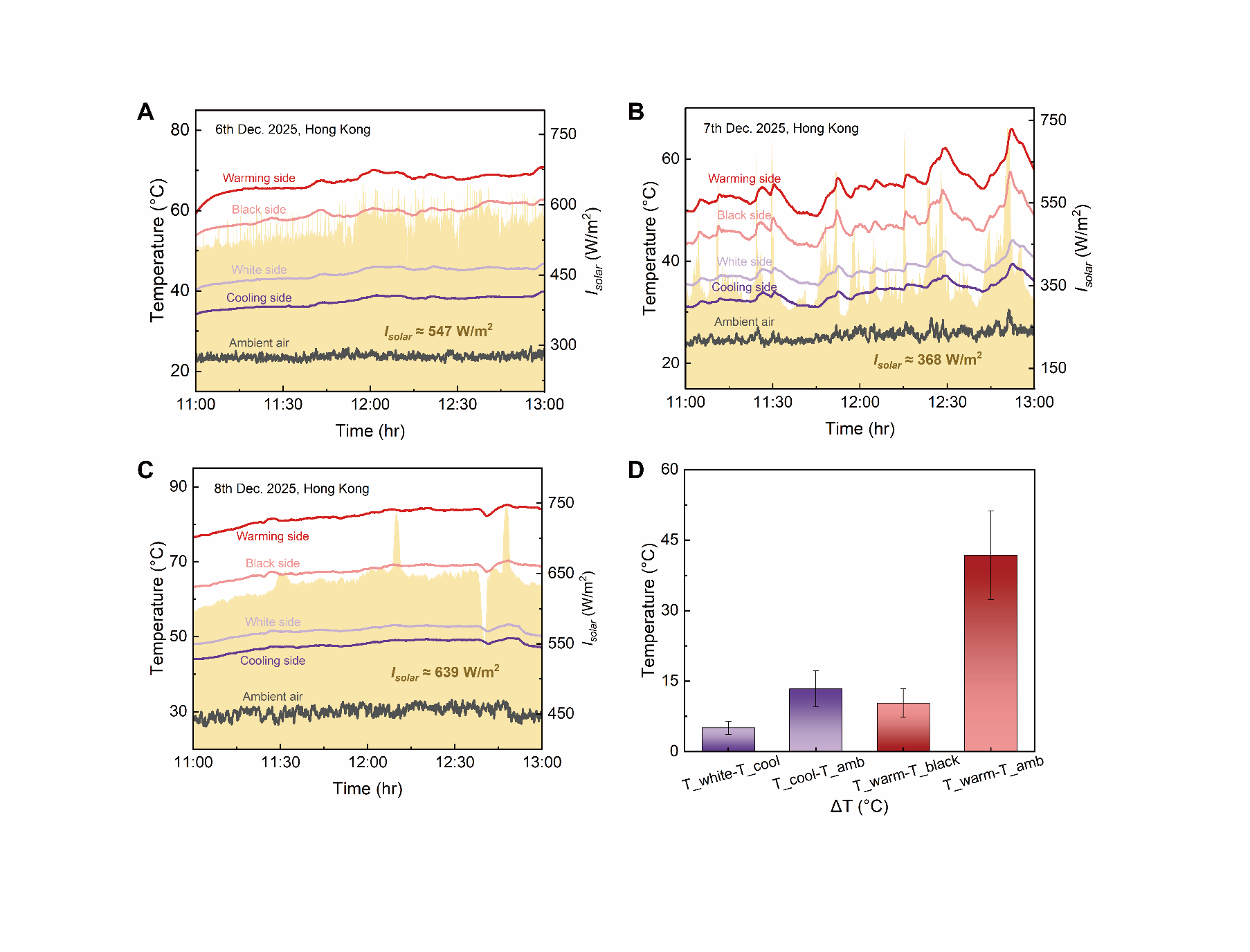


Fig. S18. (A-C) Real-time tracking of solar irradiance, ambient temperature, and thermocouple temperatures at different states. (D) The temperature difference (ΔT) between DRF and white/black fabric, and ambient air. T_white_, T_black_, T_cool_, T_warm_, and T_amb_, refer to the temperature of white fabric, black fabric, DRF in cooling and warming mode, and ambient air, respectively.


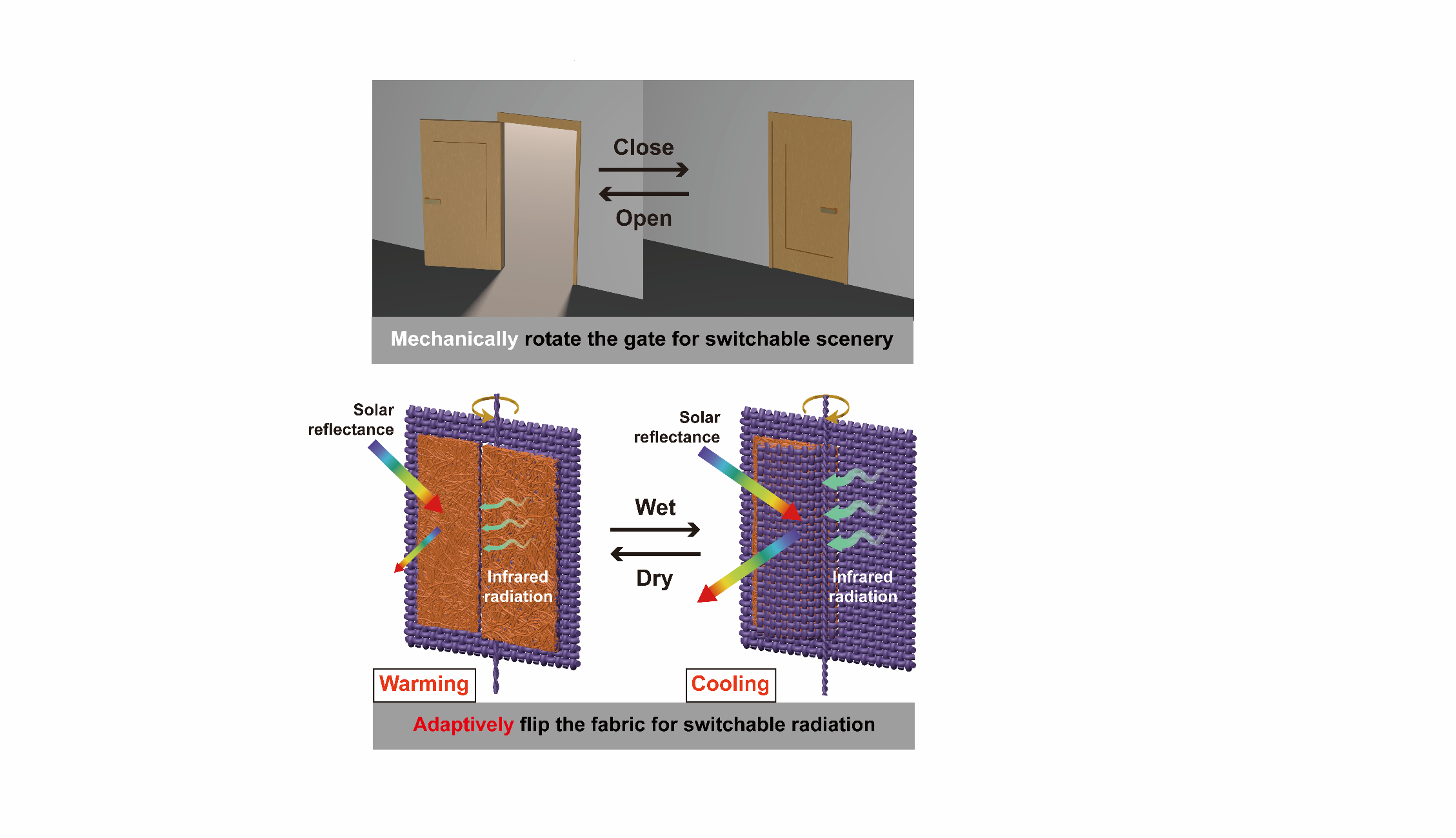


Fig. S19. Schematic illustration of adaptive and switchable and cooling inspired by a spindle-driven gate.


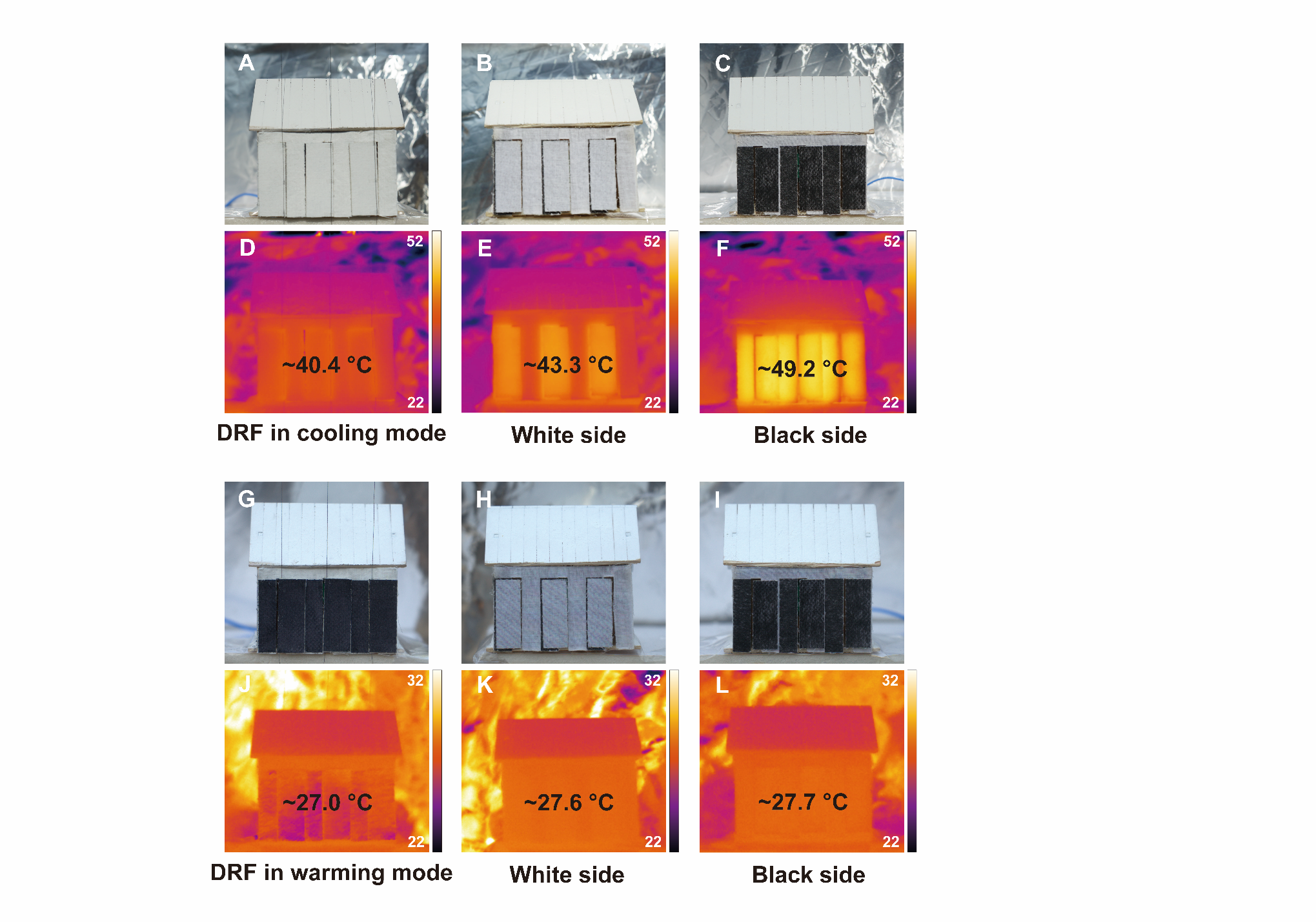


Fig. S20. Photos and thermal images of DRF (A, D, G, J), white side (B, E, H, K), and black side (C, F, I, L) of pristine fabric during midday (top) and evening (bottom).


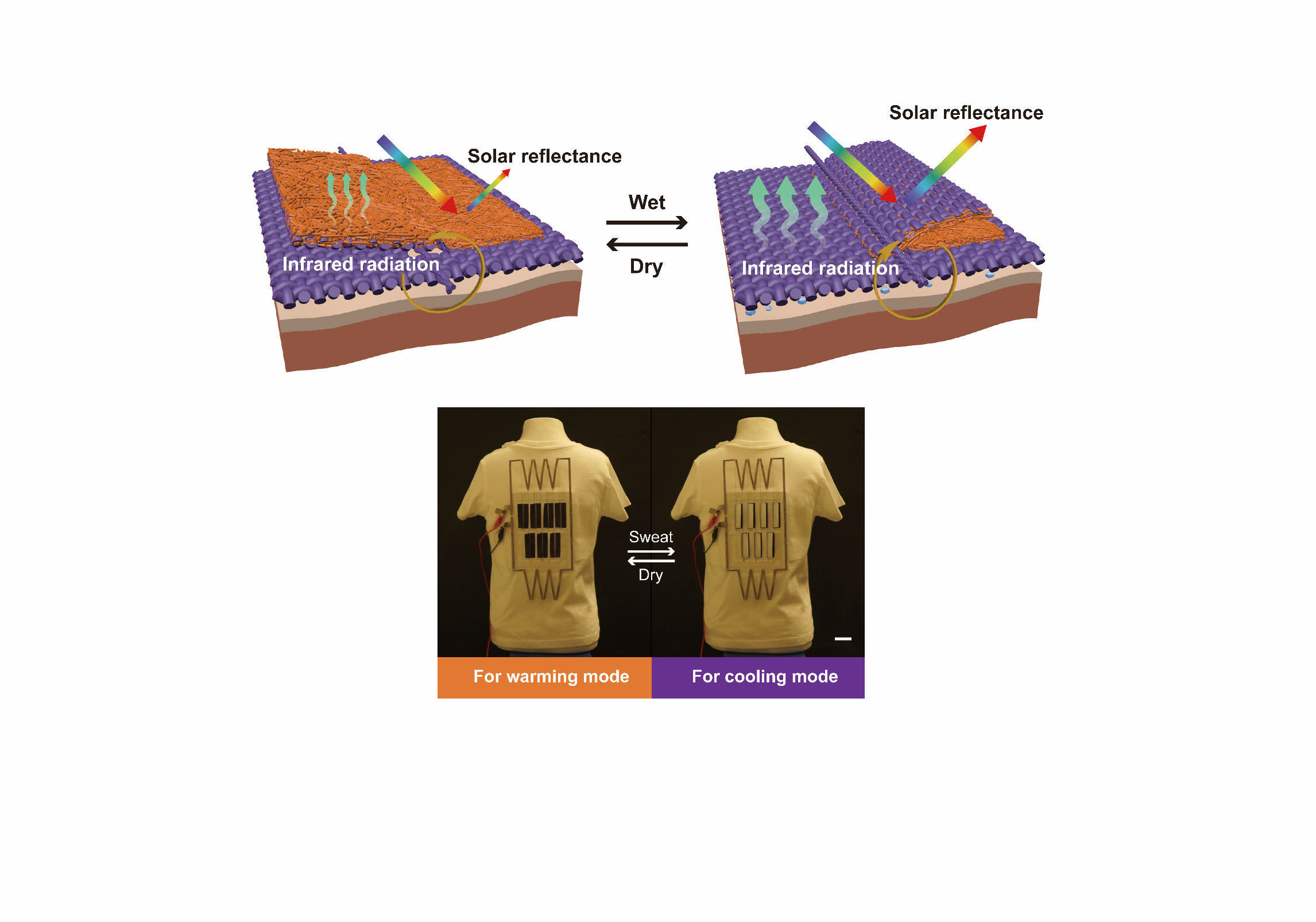


Fig. S21. Potential application in moisture-responsive thermal management clothing (scale bars = 3 cm) for warming in dry conditions and cooling in humid conditions.

Table S1. Comparisons among the HYAM and previously reported yarn muscles.

| Ref. | Yarn muscle | Radius (μm) | Max. stroke (°/cm) | Normalized Max. stroke (°) | Max. actuation speed (r.p.m.) | Normalized Max. speed (r.p.m.) | Stimuli |
| --- | --- | --- | --- | --- | --- | --- | --- |
| ^[1]^ | Silk fiber | 9.5 | 5470 | 5.2 | 1125 | 1.07 | Moisture |
| ^[2]^ | Human hair | 50 | 1224 | 6.12 | 60 | 0.30 | Moisture |
| ^[3]^ | Lotus fiber | 55 | 2000 | 11 | 200 | 1.10 | Moisture |
| ^[4]^ | Wool fiber | 20 | 1020 | 2.04 | 60 | 0.12 | Moisture |
| ^[5]^ | Viscose fiber | 9.5 | 1752 | 1.66 | 2100 | 2.00 | Water droplet |
| ^[6]^ | Spider silk | 1.5 | 2550 | 0.38 | 5.8 | <0.01 | Moisture |
| ^[7]^ | SA/GO fiber | 100 | 1500 | 15 | 200 | 1.00 | Moisture and light |
| This work | Viscose fiber | 29 | 2394 | 6.94 | 595 | 1.73 | Moisture |

Table S2. Comparisons among the dual-responsive HYAM, PYAM, SHYAM, and HYAM in this work.

| Yarn muscle | Stroke  (°/cm) | Actuation speed (r.p.m.) | Recovery speed (r.p.m.) | Actuation frequency (Hz) | Strength (MPa) |
| --- | --- | --- | --- | --- | --- |
| PYAM | 511 | 133 | 67 | ~75 °/cm stroke loss | 128 |
| SHYAM | 771 | 178 | 68 | ~84 °/cm stroke loss | 108 |
| HYAM | 1163 | 314 | 116 | 0.008 | 146 |
| HYAM (electrothermal heating) |  |  | 568 | 0.050 |  |

Movie S1. Comparison between naturally recovered and electrothermally recovered HYAM. A voltage of 15 V was supplied for electrothermal recovery.

Movie S2. The reversible moisture actuation and electrothermal recovery of HYAM on the micrometer scale.

Movie S3. Adaptive curtains based on HYAM with humidity actuation and electrothermal recovery. Each ~42 mg dual-mode fabric was driven by a ~1.01 mg HYAM (2 strands, 3000 tpm) adapted to the changing conditions. The humidity increased from ~70% to ~85%, meanwhile, the supplied voltage was 15 V.

Movie S4. An adaptive clothing based on HYAM with humidity actuation and electrothermal recovery. Each ~60.5 mg dual-mode fabric was driven by a ~2.39 mg HYAM (4 strands, 2000 tpm) adapted to the changing conditions. The humidity increased from ~70% to ~85%, meanwhile, the supplied voltage was 10 V.

References

[1] T. Jia, Y. Wang, Y. Dou, Y. Li, M. Jung de Andrade, R. Wang, S. Fang, J. Li, Z. Yu, R. Qiao, Z. Liu, Y. Cheng, Y. Su, M. Minary-Jolandan, R. H. Baughman, D. Qian, Z. Liu, Moisture sensitive smart yarns and textiles from self-balanced silk fiber muscles. *Adv. Funct. Mater.* **2019**, *29*, 1808241.

[2] X. Leng, X. Zhou, J. Liu, Y. Xiao, J. Sun, Y. Li, Z. Liu, Tuning the reversibility of hair artificial muscles by disulfide cross-linking for sensors, switches, and soft robotics. *Mater. Horizons* **2021**, *8*, 1538.

[3] Y. Wang, Z. Wang, Z. Lu, M. Jung De Andrade, S. Fang, Z. Zhang, J. Wu, R. H. Baughman, Humidity- and water-responsive torsional and contractile lotus fiber yarn artificial muscles. *ACS Appl. Mater. Interfaces* **2021**, *13*, 6642.

[4] J. Hu, M. Irfan Iqbal, F. Sun, Wool can be cool: water-actuating woolen knitwear for both hot and cold. *Adv. Funct. Mater.* **2020**, *30*, 2005033.

[5] Y. Peng, F. Sun, C. Xiao, M. I. Iqbal, Z. Sun, M. Guo, W. Gao, X. Hu, Hierarchically structured and scalable artificial muscles for smart textiles. *ACS Appl. Mater. Interfaces* **2021**, *13*, 54386.

[6] D. Liu, A. Tarakanova, C. C. Hsu, M. Yu, S. Zheng, L. Yu, J. Liu, Y. He, D. J. Dunstan, M. J. Buehler, Spider dragline silk as torsional actuator driven by humidity. *Sci. Adv.* **2019**, *5*, eaau9183.

[7] H. Liu, H. Luo, J. Huang, Z. Chen, Z. Yu, Y. Lai, Programmable water/light dual-responsive hollow hydrogel fiber actuator for efficient desalination with anti-salt accumulation. *Adv. Funct. Mater.* **2023**, *33*, 2302038.
